# Analysis of biomarker utility using a PBPK/PD model for carbaryl

**DOI:** 10.3389/fphar.2014.00246

**Published:** 2014-11-18

**Authors:** Martin B. Phillips, Miyoung Yoon, Bruce Young, Yu-Mei Tan

**Affiliations:** ^1^National Exposure Research Laboratory, US Environmental Protection AgencyDuluth, MN, USA; ^2^Institute for Chemical Safety Sciences, The Hamner Institutes for Health Sciences, Research Triangle ParkNC, USA; ^3^Bayer CropScience, Research Triangle ParkNC, USA; ^4^National Exposure Research Laboratory, US Environmental Protection Agency, Research Triangle ParkNC, USA

**Keywords:** carbaryl, PBPK, biomarkers, biomarkers of exposure, biomarkers of effect, computational toxicology

## Abstract

There are many types of biomarkers; the two common ones are biomarkers of exposure and biomarkers of effect. The utility of a biomarker for estimating exposures or predicting risks depends on the strength of the correlation between biomarker concentrations and exposure/effects. In the current study, a combined exposure and physiologically-based pharmacokinetic/pharmacodynamic (PBPK/PD) model of carbaryl was used to demonstrate the use of computational modeling for providing insight into the selection of biomarkers for different purposes. The Cumulative and Aggregate Risk Evaluation System (CARES) was used to generate exposure profiles, including magnitude and timing, for use as inputs to the PBPK/PD model. The PBPK/PD model was then used to predict blood concentrations of carbaryl and urine concentrations of its principal metabolite, 1-naphthol (1-N), as biomarkers of exposure. The PBPK/PD model also predicted acetylcholinesterase (AChE) inhibition in red blood cells (RBC) as a biomarker of effect. The correlations of these simulated biomarker concentrations with intake doses or brain AChE inhibition (as a surrogate of effects) were analyzed using a linear regression model. Results showed that 1-N in urine is a better biomarker of exposure than carbaryl in blood, and that 1-N in urine is correlated with the dose averaged over the last 2 days of the simulation. They also showed that RBC AChE inhibition is an appropriate biomarker of effect. This computational approach can be applied to a wide variety of chemicals to facilitate quantitative analysis of biomarker utility.

## Introduction

A biomarker is a substance that can be measured in an accessible biological sample and is correlated to some metric or condition of interest in the body. Examples of accessible biological samples include urine, blood, saliva, and hair; conditions of interest include disease conditions, clinical states, evidence or extent of exposure, and manifestation of biological effect. In environmental sciences, two categories of biomarkers are widely used: biomarkers of exposure and biomarkers of effect (WHO/IPCS, 1993; IUPAC, 2004). “Biomarkers of exposure” are markers that infer exposures to xenobiotics. They may be the parent chemical itself, a metabolite, or an endogenous substance; at minimum, the marker must exhibit a predictable relationship in response to exposure (USEPA, 2014). In some cases, this predictable relationship is strong enough to reconstruct exposures from measured biomarker concentrations. In the majority of cases, however, this relationship only allows for qualitative assessment such as trend analysis. “Biomarkers of effect” are markers that are either known to be directly associated with specific adverse outcomes (e.g., cholinergic poisoning, Kim et al., 2010; Marsillach et al., 2011), or to be empirically associated with particular systemic effects (e.g., oxidative stress, Peluso et al., 2013; Zhang et al., 2013). Biomarkers of effect may be directly involved in the mode of action, which describes the sequence of key events that link some elevated tissue doses to an adverse toxicological and/or clinical effect. The general distinctions between biomarkers of exposure and biomarkers of effect are not always exclusive; some biomarkers may fall into both categories. For example, red blood cell (RBC) cholinesterase inhibition has been considered to be both a biomarker of exposure to organophosphate or carbamate pesticides and an early biomarker of effect on inhibition of brain cholinesterase activity (ATSDR, 1997; Garabrant et al., 2009).

Technological and scientific advances in analytical and clinical chemistry have resulted in increased collection, analysis, and reporting of human biomarkers in targeted cohort studies or ongoing national surveys (e.g., the National Health and Nutrition Examination Survey [NHANES] in the United States). There is increased interest in utilizing these biomarker data to characterize/estimate exposures or to correlate them to health outcomes in epidemiological studies. These applications are beyond the traditional uses of biomarkers, e.g., observing trends over time and across different populations. As new areas of applications are explored, it is critical to evaluate the utility of a specific biomarker measurement for a particular purpose, either to reflect exposures to a chemical or to predict an association between exposures and an adverse effect. Computational modeling is one of the tools that can assist in evaluating the appropriateness of a biomarker's utility for a specific purpose.

In the current study, a physiologically based pharmacokinetic/pharmacodynamic (PBPK/PD) model for carbaryl (Yoon et al., 2012, 2014) was used to evaluate various types of biomarkers for their utility as markers of external exposure or markers of early effects. This PBPK/PD model quantitatively connects external exposure (i.e., time course of oral exposure events) to the concentration of the active species at the target organ (i.e., carbaryl in brain), which in turn is connected to known key events along the mode of action (e.g., acetylcholinesterase [AChE] inhibition in RBC and in brain). In addition, the model predicts the urinary concentration of the principal metabolite, 1-naphthol (1-N), which has been used as a biomarker of exposure for carbaryl (Meeker et al., 2007). This model was chosen for our study for several reasons. First, carbaryl is a well-studied carbamate pesticide and its mechanism of action is generally agreed upon (Carlock et al., 1999). Second, the PBPK/PD model for carbaryl has the ability to predict both biomarkers of exposure and biomarkers of effect. Third, this model includes sufficient complexity (e.g., metabolism, urinary excretion) to highlight the challenges in selecting an appropriate biomarkers for non-persistent chemicals, while being simple enough to allow for relatively straightforward analysis.

The PBPK/PD model was used to simultaneously track various types of biomarkers and tissue concentrations as a function of exposure doses, so that interdependent relationships between exposure/effects and biomarkers could be examined in depth. (Note: Time course data for a single simulated individual may be found in Supplementary Figure 1). More specifically, the simulated biomarkers (e.g., concentrations of carbaryl and 1-N in tissues or urine) were analyzed for correlations between external exposure concentrations or brain cholinesterase inhibition, using linear regression analysis, to determine their utility as markers for either exposure or biological effects. The objective of this study is to demonstrate the use of computational models to gain quantitative insight into the utility of biomarkers for estimating exposure and early biological events. Our approach to would be applicable to other chemicals and will contribute to expanding the utility of biomarkers beyond their traditional uses.

## Methods

The PBPK/PD model for carbaryl describes the disposition of carbaryl and 1-N, the binding of carbaryl to cholinesterases in blood and brain, and the urinary excretion of 1-N. The structure of the model along with *in vitro*-based parameterization of the model were described in detail in a previously published study (Yoon et al., 2012). For this study, the model code was translated into MATLAB® (R2013b version 8.2.0.701, MathWorks, Natick, MA). The MATLAB code may be found in the Supplementary Material. The m-files are available from the authors upon request.

The model has six compartments: GI tract, liver, fat, brain, blood (which is further subdivided into plasma and RBC sub-compartments), and a compartment for the rest of the body. All tissue compartments were described in the model as diffusion-limited. Absorption was included by adding the oral dose directly to the GI tract compartment. Distribution was from the GI tract to the liver and then to the blood. The blood compartment communicated with the liver, brain, fat, and “rest of body” compartments. Metabolism described in the model included hydrolysis to 1-naphthol (which takes place in all compartments) and metabolism of 1-naphthol to “other metabolites,” which are not explicitly defined. The pharmacodynamic sub-models in the brain and RBC compartments described the synthesis and degradation of AChE, as well as binding of carbaryl to the AChE protein and release of the decarbamylated 1-naphthol from the protein. 1-Naphthol was eliminated in the urine.

**Figure 1:**
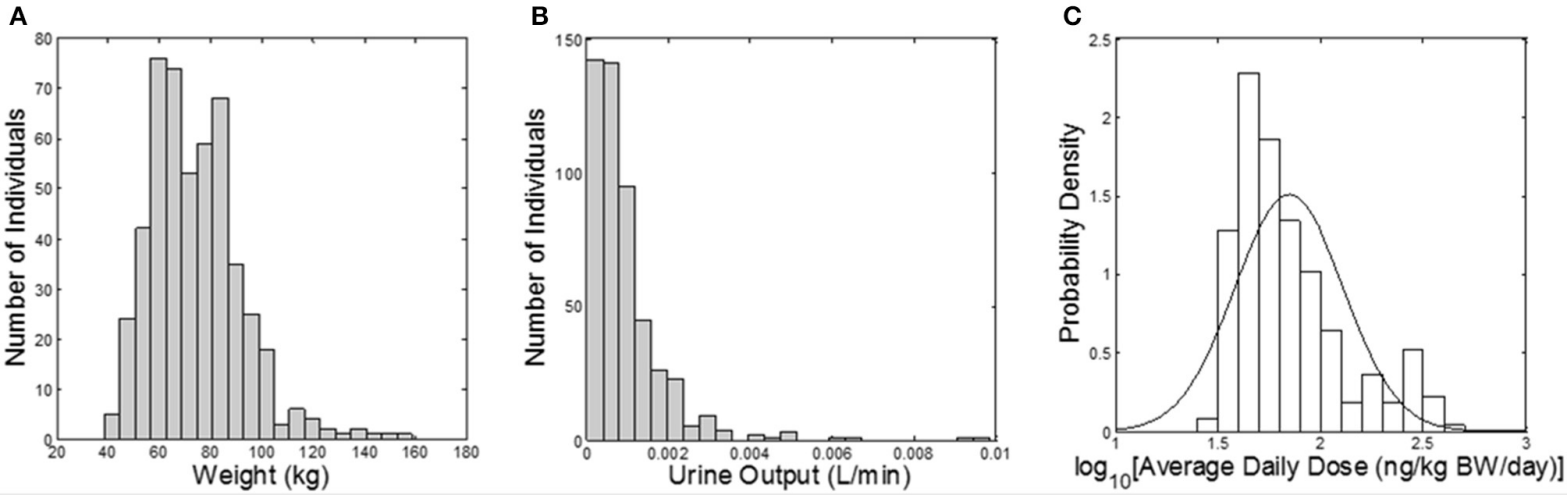
**Model inputs from CARES and NHANES**. **(A)** Histogram of subject body weights (kg); **(B)** histogram of urine flow rates (L/min) assigned from NHANES; **(C)** probability density of the daily dose of carbaryl averaged for each subject over the year (ng/kg BW/day) from CARES (solid line shows the fitted distribution; geometric mean: 70.2 ng/kg/day, geometric standard deviation: 1.84).

The Cumulative and Aggregate Risk Evaluation System (CARES®, production version 3.0 build 1.3.4, ILSI Research Foundation, Washington, D.C.) was used to generate a “virtual” population of 500 individuals (based on the 1990 U.S. Census data), between age 20 and 90 years old; and CARES was used to estimate exposure to carbaryl via food and water (based on the US Department of Agriculture's 1994–1996, 1998 Continuing Survey of Food Intake by Individuals). The output of the CARES model includes the age, gender, body weight, and within-day exposure profiles (time vs. carbaryl intake doses) for each individual for 365 days. Urine output values (the volume of a urinary void divided by the time between voids, a measure of the rate of bladder filling) were randomly sampled from the NHANES 2009–2010 dataset (CDC, 2014a). The urine output value is necessary to convert moles of 1-naphthol eliminated in the urine (the output of the simulation) into molar concentrations, as discussed further below. CARES-simulated body weights and exposure profiles, along with the NHANES urine output values, served as model inputs (Figure 1).

The PBPK/PD model was used to predict the time courses of (1) a biomarker of exposure (as reported in NHANES 2007–2008 [CDC, 2014b]): urinary 1-N concentrations in spot samples; (2) a potential biomarker of exposure: plasma carbaryl concentrations in spot samples; (3) a biomarker of early effect: RBC AChE inhibition; and (4) two inaccessible dose metrics in the target tissue: 24-h average carbaryl concentrations in brain and brain AChE inhibition. One week of exposure was simulated, with as few as one and as many as 23 exposure events per day (median of four per day) based on the CARES data. To simulate the spot sampling of biomarkers in plasma or urine, sampling times were selected from the final 24 h of the simulation using a uniform distribution. For 1-N in urine, the time of the most recent void was simulated from the same 24 h period, with the constraint that it must be >1 h earlier than the sampling time. The spot urinary 1-N concentration was calculated using the following equation:

$$\text{Spot Biomarker}=\frac{moles_{\text{sampling}}-moles_{\text{mrv}}}{\left(t_{\text{sampling}}-t_{\text{mrv}}\right)\times \text{urine output}}$$

where “Spot Biomarker” is the spot biomarker concentration of 1-N in the urine (nM), *moles*_sampling_ is the cumulative amount of 1-N excreted by the time of sampling (nmol), *moles*_mrv_ is the cumulative amount of 1-N excreted by the time of the most recent void (nmol), *t*_sampling_ is the time of sampling (min), *t*_mrv_ is the time of the most recent void (min), and “urine output” is the value for the accumulation of urine in the bladder selected from the NHANES 2009-2010 report.

A sensitivity analysis was performed on all 62 model parameters for five different model outputs: amount of urinary 1-N (μmol), depression of brain AChE activity (%), depression of red blood cell AChE activity (%), amount of carbaryl in brain tissue (μmol), and concentration of carbaryl in blood plasma (μM) (see Supplementary Tables 1, 2). A parameter was considered to be sensitive if its normalized sensitivity coefficient was >0.1. Normalized sensitivity coefficients were calculated as described elsewhere (Peters, 2012). The sensitivity analysis was repeated at three dose levels: the lowest average daily dose (29.7 ng/kg/day), the highest average daily dose (426 ng/kg/day), and 1000 × the highest average daily dose (426,000 ng/kg/day). No difference was identified in those parameters that were found to be sensitive at the three doses (Supplementary Table 1 contains a spreadsheet listing which parameters were found to be sensitive for which endpoints). Subsequently, a Monte Carlo analysis was conducted to vary each sensitive parameter, as well as the time of sampling (*t*_sampling_) and time of most recent void (*t*_MRV_). Supplementary Table 2 lists the central tendency (mean or geometric mean), distribution width (standard deviation or geometric standard deviation), and distribution type (normal or lognormal) that was used for Monte Carlo analysis. All 500 individuals from CARES were run 10 times each, which resulted in 5000 iterations for the Monte Carlo analysis. Body weights, urine outputs, and exposure profiles were the same for each individual over the 10 runs.

To determine the utility of various biomarkers to reflect exposure concentrations or early biochemical changes, four sets of linear regression analyses were conducted in this study.

1. Examine the correlations between urinary 1-N concentrations from spot samples and daily doses averaged over four exposure periods to determine the appropriate time frame of exposures that is most relevant to the measurement of 1-N in urine. The four exposure periods examined were the prior year, the prior week, the prior 2 days, and the prior 24 h.
2. Compare the correlations between six model-output variables and the average daily dose over the last 2 days of exposure (ng/kg/day). These six variables are spot 1-N concentrations in urine (a biomarker of exposure); 24-h averaged carbaryl concentrations in blood (target tissue dose); spot and 24-h averaged % AChE inhibition in RBC (an early biochemical change); spot and 24-h averaged % AChE inhibition in brain (a biochemical change).
3. Compare the correlations between 24-h averaged % AChE inhibition in RBC/brain with average daily doses, limiting the dataset to only average daily doses over the last 2 days of exposure that were >50 ng/kg/day.
4. Examine the correlations between spot or 24-h averaged % AChE inhibition in brain and four model-output variables to determine which potential biomarker best reflects the changes of AChE in brain. These four variables were spot and 24-h averaged % AChE inhibition in RBC; spot 1-N concentrations in urine; and spot carbaryl concentrations in plasma.
5. Compare the correlations between 24-h average brain concentration of carbaryl (target tissue dose) and two potential biomarkers of exposure: spot 1-N concentrations in urine and spot carbaryl concentrations in plasma.

Statistical analysis and graphics were produced using MATLAB® and Microsoft Office Excel® (version 2007, Microsoft Corporation, Redmond, WA).

## Results

**Figure 2:**
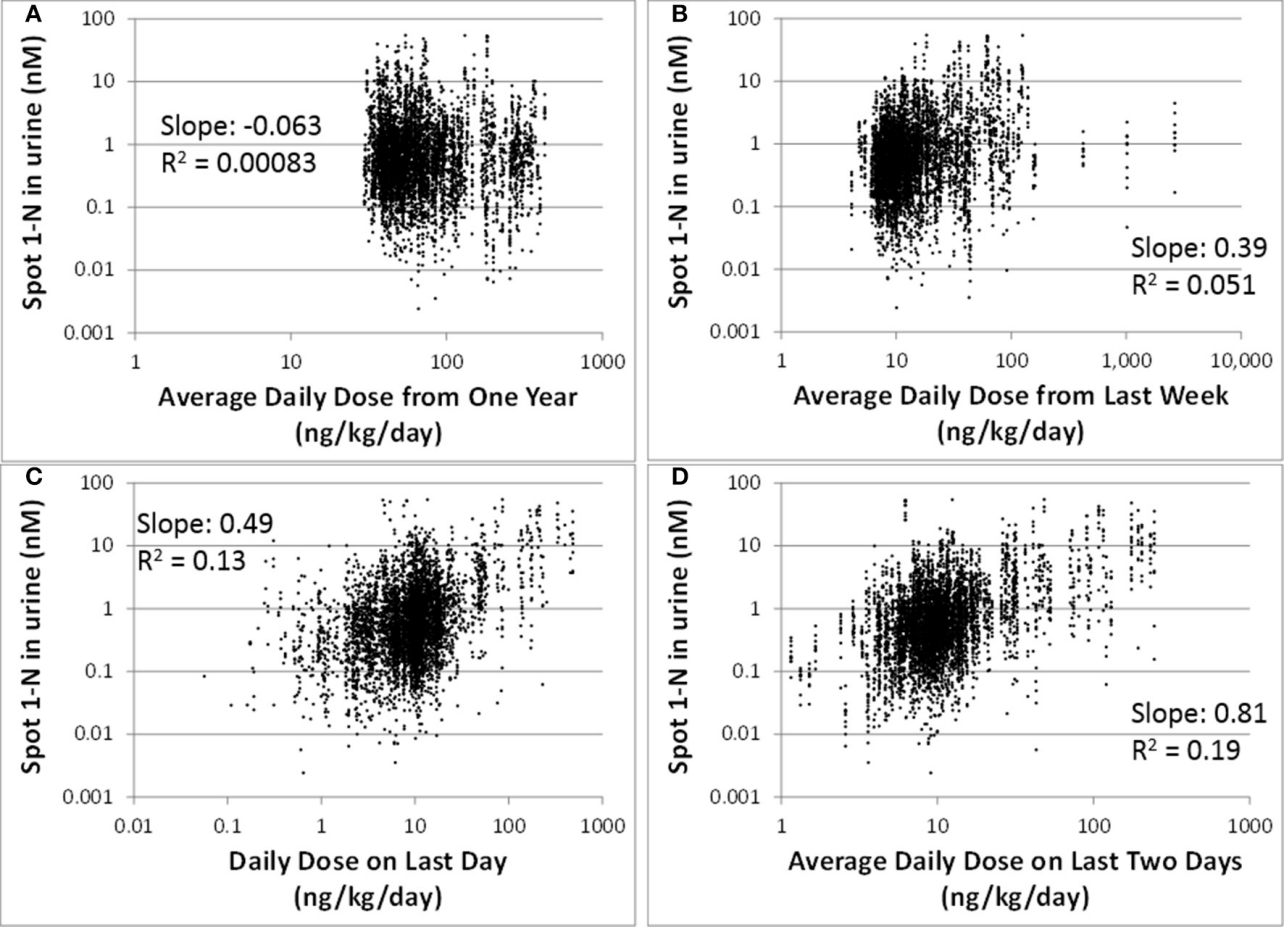
**Spot 1-N concentrations in urine (nM) vs. average daily doses from four exposure periods (ng/kg/day) for 5000 simulations**. **(A)** Exposure doses averaged over the past year. **(B)** Exposure doses averaged over the past week. **(C)** Exposure dose averaged over the 24 h prior to sampling. **(D)** Exposure dose averaged over the past 2 days.

Our regression analysis results showed that spot urinary 1-N concentrations (nM) had no correlation with averaged doses (ng/kg/day) from the past year (slope = −0.063, *R*^2^ = 0.00083), the past week (slope = 0.39, *R*^2^ = 0.051), or the past 24 h (slope = 0.49, *R*^2^ = 0.13) (Figures 2A–C). The best exposure period reflected by spot 1-N concentrations in urine was the average daily dose from the past 2 days (slope = 0.81, *R*^2^ = 0.19) (Figure 2D).

**Figure 3:**
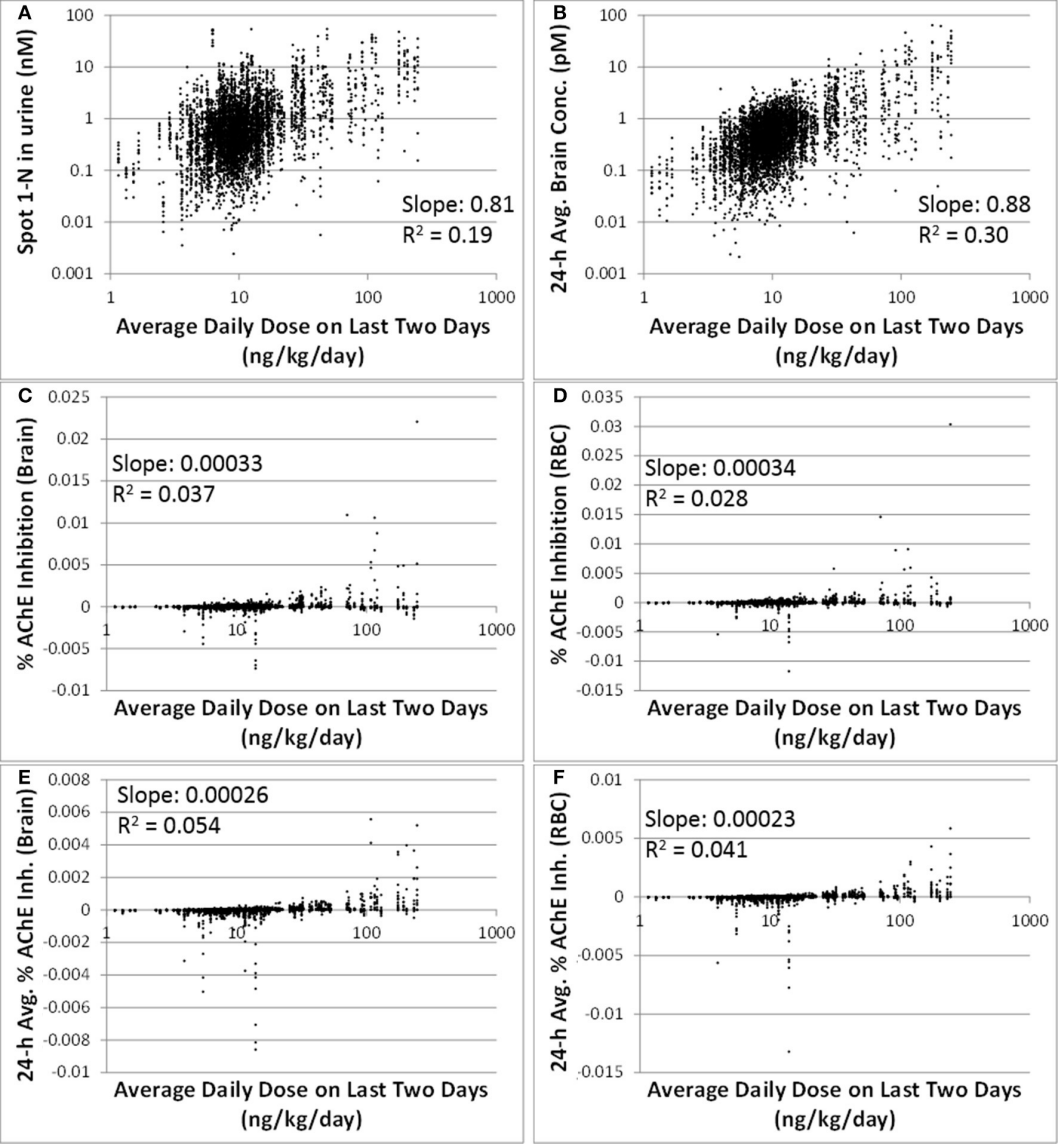
**Correlations between six model-output variables and the average daily doses over the last 2 days of exposure (ng/kg/day) for 5000 simulations**. **(A)** biomarker of exposure: spot 1-N in urine (nM); **(B)** target tissue concentration: carbaryl concentrations in brain averaged over the 24 h prior to sampling (pM); **(C)** Biomarker of early biochemical changes at the target tissue: percent AChE inhibition in brain tissue (baseline = 0%); **(D)** Biomarker of early biochemical changes at peripheral tissue: percent AChE inhibition in red blood cells (baseline = 0%); **(E)** Percent AChE inhibition in brain tissue averaged over the last 24 h prior to the urine sampling time; **(F)** Percent AChE inhibition in red blood cells averaged over the last 24 h prior to the urine sampling time.

**Figure 4:**
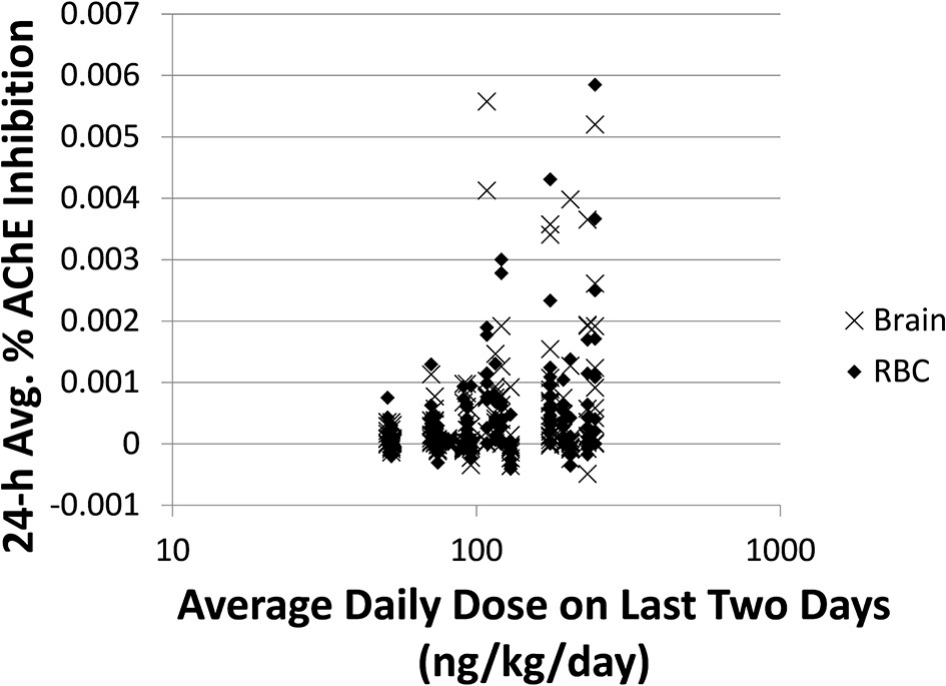
**Time-averaged (over 24 h) % AChE inhibition for brain (x) and RBC (♦) vs. average daily doses ≥50 ng/kg/day from the last 2 days for 5000 simulations**. For brain: slope = 0.0012, *R*^2^ = 0.089. For RBC: slope = 0.0011, *R*^2^ = 0.096.

Comparing to spot 1-N concentrations in urine (nM) (slope = 0.81, *R*^2^ = 0.19), 24-h averaged carbaryl concentrations in brain (pM) had a stronger correlation (slope = 0.88, *R*^2^ = 0.30) with the average daily doses (ng/kg/day) from the past 2 days (Figures 3A,B). Spot % AChE inhibition in brain (slope = 0.00033, *R*^2^ = 0.037) and in RBC (slope = 0.00034, *R*^2^ = 0.028) had no correlation with the average daily doses (ng/kg/day) from the past 2 days (Figures 3C,D). While there appeared to be no correlations between 24-h averaged % AChE inhibition in brain (slope = 0.00026, *R*^2^ = 0.054) and in RBC (slope = 0.00023, *R*^2^ = 0.041) and the average daily doses (ng/kg/day) from the past 2 days (Figures 3E,F), it was interesting to see that when the analysis was limited to those individuals with average daily doses higher than 50 ng/kg/day, a positive correlation emerged between 24-h averaged % AChE inhibition (for brain, slope = 0.0012, *R*^2^ = 0.089; for RBC, slope = 0.0011, *R*^2^ = 0.096) and average daily doses from the past 2 days (Figure 4).

**Figure 5:**
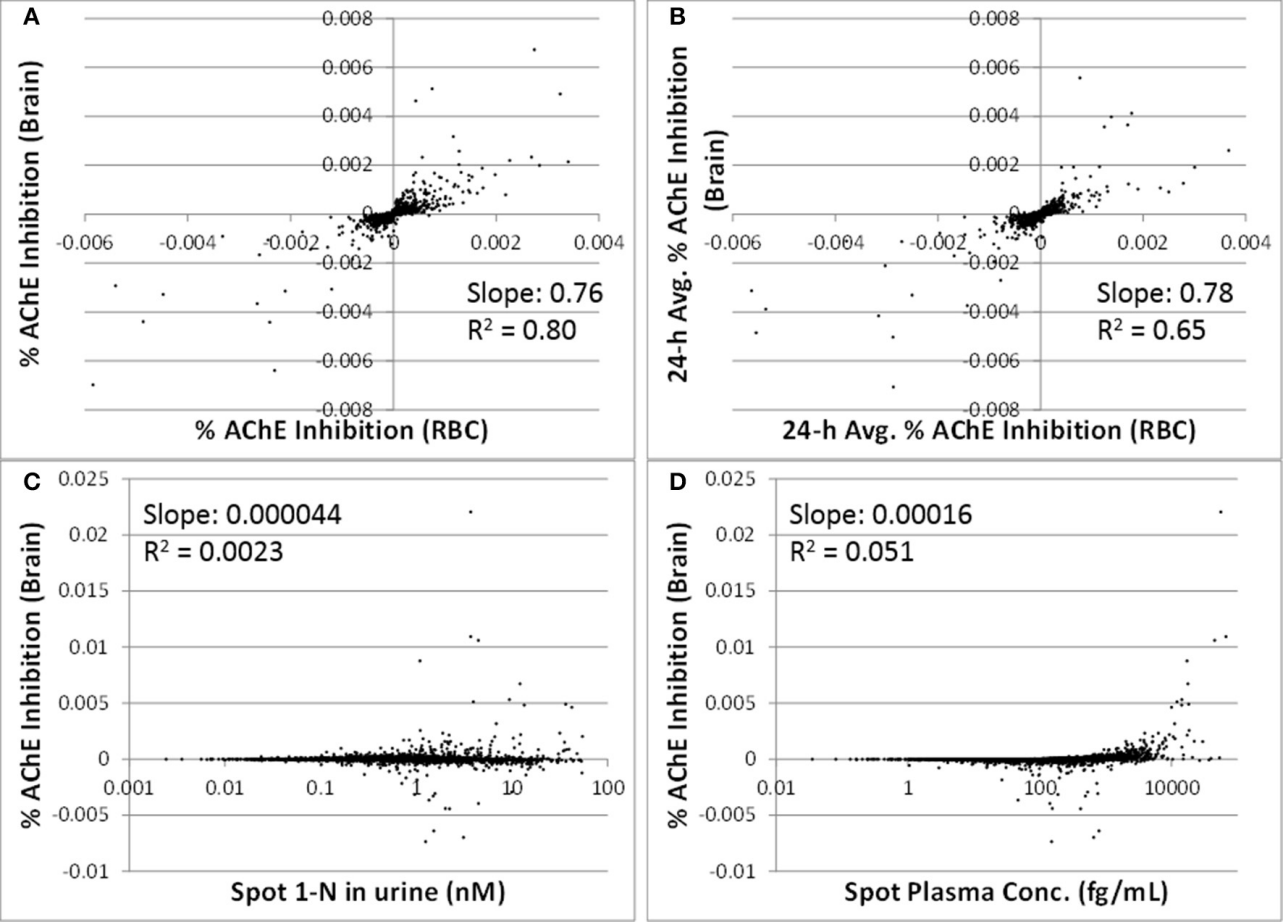
**Utility of peripheral markers to predict inhibition in the target tissue (brain) for 5000 simulations**. **(A)** Spot percent AChE inhibition in the brain vs. spot percent AChE inhibition in red blood cells, taken at the same time as the urine sampling; **(B)** 24-h averaged AChE inhibition in brain vs. 24-h averaged AChE inhibition in RBC (time averaging was over the 24 h prior to urine sampling); **(C)** Spot percent AChE inhibition in the brain vs. spot 1-N concentrations in urine (nM); **(D)** Spot percent AChE inhibition in the brain vs. spot carbaryl concentrations in plasma (pM).

The correlations between spot or 24-h average % AChE inhibition in brain and in RBC were strong (slopes were > 0.76, R^2^ were > 0.65) (Figures 5A,B). Spot % AChE inhibition in brain had no correlation with spot 1-N concentrations in urine or spot carbaryl concentrations in plasma (slopes were < 0.0002, R^2^ were < 0.06) (Figures 5C,D).

**Figure 6:**
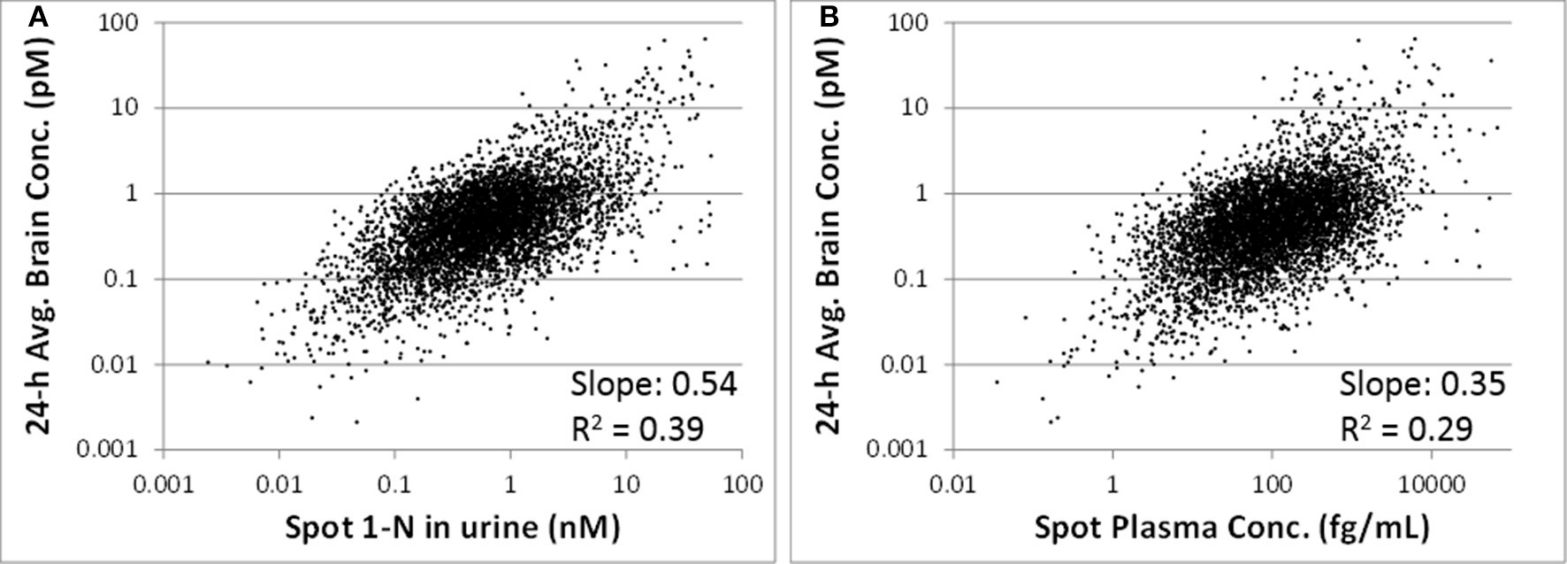
**(A)** Utility of spot 1-N concentrations in urine to predict the concentration of carbaryl in the target tissue (brain) for 5000 simulations. **(B)** Utility of spot carbaryl concentrations in brain to predict the concentration of carbaryl in the target tissue (brain).

The 24-h average brain concentration is a measure of the “target tissue dose.” Spot 1-N concentrations in urine (slope = 0.54, *R*^2^ = 0.39) were more strongly correlated to the 24-h brain carbaryl concentration than spot carbaryl plasma concentrations were (slope = 0.35, *R*^2^ = 0.29) (Figure 6). The correlation between spot 1-N concentrations in urine and 24-h averaged carbaryl concentrations in brain (Figure 6A) was stronger than its correlation with average daily doses of carbaryl (Figure 2A).

## Discussion

Biomarkers and biomonitoring are promising tools to link different elements along the source-to-outcome continuum for the purpose of understanding the public health implications of exposure to environmental chemicals (Sobus et al., 2011). Biomarkers of exposure infer exposures to exogenous chemicals, and can be used to complement environmental or personal monitoring (USEPA, 2014). They can be useful in estimating variability in exposures within a population or comparing groups of individuals. Biomarkers of effect signify biological responses to chemical exposures, and can be used to investigate chemical toxicity or changes in biological functions (USEPA, 2014). Despite the potential utility in providing robust assessment of exposures or health risks, there exists a need for developing methods to evaluate whether a biomarker could be a marker for predicting either exposure or effects. Two of the key considerations in evaluating the utility of a biomarker to assess exposures or effects are (1) accounting for absorption, distribution, metabolism, and excretion (ADME) characteristics and (2) estimating the degree of correlation between the biomarker and the metric of interest.

In order to use biomarkers in any application, accessibility and limit of detection (LOD) should always be considered first. A biomarker must be sampled from an accessible biological matrix, and the most common matrices are urine and blood. Depending on the pharmacokinetic properties of a chemical, i.e., ADME characteristics, other matrices may need to be considered to obtain a more “appropriate” biomarker for estimating exposures or risks (Calafat and Needham, 2008). For example, some chemicals lack any measurable excretion in urine or do not build up to appreciable levels in blood due to their distribution characteristics to peripheral compartments (e.g., highly fat-soluble compounds), rapid elimination (e.g., volatile organic compounds), or poor absorption (e.g., Olestra). For these chemicals, biomarkers measured in hair, saliva, nail clippings, breast milk, or breath may correlate better with exposure concentration and/or magnitude of effect. The detection limit can also have an important impact on biomarker utility. Improvements in analytical capability would be expected to lower the detection limit toward zero, but never to zero. It is problematic to interpret biomarkers measurements that contain a large number of non-detects (e.g., *o*-phenylphenol, CDC, 2014a), either in the context of exposure or health risks. Two common approaches for analyzing such data is to truncate the distribution of biomarker concentrations at the LOD, or to replace non-detects with some value relevant to the LOD (e.g., half of the LOD). It is important to bear in mind that these approaches would suggest that the non-detects are missing values or uninformative data rather than true values indicative of low- or non-exposure. Computational studies skirt this issue somewhat since very small values can still be predicted using the equations, and there is no “detection” required. In addition, computational simulations can be used to explore the highest exposure concentrations correlated to a biomarker concentration at the LOD. Due to physiological differences and variability in the timing between the exposure event(s) and biomarkers sampling, many different levels of exposure can lead to the same biomarker concentration (Grulke et al., 2014). If exposure at a dangerous level could potentially lead to a biomarker concentration below the LOD, it could suggest that a better analytical method is necessary.

ADME characteristics determine the quantitative relationship that links the magnitude of exposures to biomarker levels, and from there to the magnitude and incidence of adverse outcomes at biological endpoints. For the example of carbaryl, the parent compound is responsible for inhibiting cholinesterase and the accessible biomarker is a urinary metabolite. The urinary biomarker concentration is somewhat directly proportional to the exposure, but its relationship to the effect (i.e., inhibition of AChE in brain) may be directly or inversely proportional, depending upon the relative rates of metabolism and urinary excretion. If an individual has higher rate of metabolism from carbaryl to 1-N, or higher rate of excretion of 1-N to urine, s/he may have a higher urinary 1-N concentration but a lower carbaryl concentration, and therefore a lower level of AChE inhibition in the brain. On the other hand, if most people in a population have similar rates of metabolism and excretion, higher urinary 1-N concentrations may suggest higher exposure concentrations and higher carbaryl concentrations, and therefore greater inhibition of AChE in the brain.

The degree of correlation between biomarkers and exposure or effects is often difficult to determine because such analysis requires *de novo* experiments specifically designed for this purpose. In reality, biomarker measurements are rarely collected in conjunction with other exposure-related data. There exists a need for systematically integrating various types of biomarkers with other knowledge (e.g., ADME characteristics) to better inform effects of exposure in the interest of promoting public health. One of the most powerful techniques for integrating disparate classes of knowledge is computational modeling (Sohn et al., 2004; Georgopoulos et al., 2009; Mosquin et al., 2009; Phillips et al., 2014). In the current study, a linked CARES-PBPK/PD model was used to capture the dynamic relationships between exposure, tissue concentrations, metabolism, biomarker concentrations in various matrices, and early biological effects. This modeling approach provided an unparalleled capability to simulate chemical concentrations at any arbitrary time point, allowing correlations between various metrics to be thoroughly explored. Through this simulation process, biomarkers with the greatest predictive or discriminatory power were identified to link to exposure or biological effects, providing valuable insight into the utility of biomarkers for different purposes.

In the current study, linear regression analysis was performed to investigate the correlations between biomarker levels and exposure concentrations or brain AChE inhibition. The results of linear regression provide a rough, yet quantitative estimation of two properties: sensitivity and variability. Here, sensitivity is not used in the typical sense of biostatistics (i.e., the rate of true positives for a binary variable), but in the sense commonly encountered in analytical chemistry: “the change in the response of a system for a small change in the stimulus causing the response” (Pardue, 1997). For highly “sensitive” biomarkers of exposure, a small change in the exposure concentration corresponds to a large change in the biomarker level. This result gives discriminatory power to distinguish high levels of exposures from low ones. Sensitivity is approximated as the slope of the regression. The slope is not only a measure of the direction of the correlation (positive or negative), but can be used to determine how useful a particular biomarker is for reconstructing exposure, predicting acetylcholinesterase inhibition, or both. A slope near zero means that the predictive value is poor, while a slope that is greater in magnitude suggests stronger potential for the use of the biomarker in a particular capacity. When the slope is near zero, it is much more difficult to accurately reconstruct exposures from biomarker data because a huge range of exposure is consistent with a single biomarker concentration. Variability is rooted in the natural differences between otherwise similar biological systems. Nothing in biology is an exact duplicate of another. When two variables are correlated (e.g., intake of carbaryl vs. 1-N in urine), natural variability may attenuate the correlation, moving the slope toward zero. In this case, the R^2^ value can be used as an indicator of the variability. Taken together, the slope and R^2^ value can provide a reasonable indication of (1) whether a correlation exists, (2) how sensitive a biomarker might be for its prospective use (e.g., reconstructing exposure or predicting adverse effects), and (3) how much variability is present.

We have intentionally avoided proposing cut-off values to demarcate “good” vs. “bad” biomarkers. Instead, we propose that the quantitative information derived from the regression analysis be used to determine the utility of biomarkers on a relative, rather than an absolute basis. For instance, urinary 1-N is a better biomarker of exposure than % RBC inhibition because its R^2^ value is 0.19 instead of 0.028 (Figure 3). If RBC inhibition data were all we had, however, then maybe it would suffice as a (rough) estimate of exposure since there is a correlation, just not a very strong one. R^2^ values are a statistical measure defined as the fraction of the response variable variation that is explained by the linear model (R^2^ = explained variation/total variation). As an example, for the correlations presented in Figure 2, an R^2^ value less than one implies that the variation in biomarker measurements cannot be explained by intake doses alone.

While linear regression may at first seem too simplistic to offer real insight, it is important to remember that this analysis would not be possible without the use of a model that allows us to match different metrics (blood concentration, urinary concentration, brain AChE inhibition, etc.) at exactly the same time points for an individual. In real life scenarios, it is prohibitively expensive to get large numbers of subjects and biological samples (such as the 500 simulated subjects in this study and a time course of blood concentrations) and unethical to perform certain experiments (such as repeated brain biopsies to estimate a 24-h average brain concentration and brain AChE inhibition). Our modeling approach, even using the simple linear regression analysis in our study, has enabled us to investigate the sensitivity of biomarkers for their intended uses, and also to explore the sources of variability (e.g., urine output) to gain insight regarding how to use these biomarkers to estimate exposure or biological effects.

Biomarkers are generally collected as a snapshot of an individual's internal (e.g., blood) or excreted (e.g., urine) doses. Thus, biomarkers of shorter half-life chemicals (e.g., phthalates) often reflect daily variation in exposure patterns and magnitudes, while biomarkers of longer half-life chemicals (e.g., mercury) tend to reflect long-term average exposures (Clewell et al., 2008; Sobus et al., 2011). Carbaryl has a relatively short half-life of 9 h (Feldmann and Maibach, 1974), so it is expected that its biomarker of exposure, 1-N in urine, reflects recent exposures. From our analysis, urinary 1-N concentrations had a very poor correlation with average doses from the past year or past week. This observation is expected because of carbaryl's short half-life. What was unexpected is that urinary 1-N concentrations had no correlation with the average intake over the final 24 h period prior to sampling; instead, the concentration of 1-N was a better marker for the average daily dose over the final 2 days (Figure 2). This finding indicates that accumulation of 1-N in urine does not only reflect the clearance of carbaryl in the body, but multiple ADME processes including the kinetic properties of metabolites. In this case, the observed correlation is likely attributable to the relatively slower excretion of 1-N compared to carbaryl.

In addition to 1-N concentrations in urine, correlations between daily intakes of carbaryl and five other model outputs were examined, including % AChE inhibition in RBC (an early biological effect), % AChE inhibition in brain (a biological effect), and 24-h averaged carbaryl concentrations in brain (target tissue dose) (Figure 3). While carbaryl in brain has a stronger correlation with average daily intake in the past 2 days when compared to 1-N in urine, this variable cannot be a biomarker of exposure since brain tissue is not readily available from living subjects. (Note: For Figure 3, the daily intakes are averaged over the final 2 days of the simulation. See Supplementary Figure 2 for the same correlations, but with the daily intake being the sum of exposures over the 24 h period prior to sampling.) Percent AChE inhibition in brain and RBC, either spot or 24-h averaged, were found to have no correlation with daily intake of carbaryl averaged over the past 2 days (Figures 2C–F). An exception was that 24-h average % AChE inhibition showed a positive correlation with carbaryl intake at levels higher than 50 ng/kg/day (Figure 4). This observation implies that the lack of correlation was caused by no inhibition of AChE at lower intake levels. The mode of action for carbaryl toxicity involves carbaryl inhibiting AChE in the brain, which then can result in cholinergic overstimulation and subsequently lead to autonomic and neuromuscular dysfunction. AChE inhibition in the brain is therefore a marker of early biochemical changes before an adverse clinical effect occurs, but this marker is not measurable in humans. In the current study, three potential biomarkers were evaluated for their utility to represent AChE inhibition in brain. Our findings (Figures 5A,B) were consistent with several studies conducted in laboratory animals where good concordance was found between brain and RBC AChE inhibition (McDaniel et al., 2007; Moser et al., 2010). This result further supports the use of RBC AChE as an ideal biomarker of early biochemical changes, especially since assays already exist to measure RBC AChE activity, and RBC AChE is generally more sensitive than brain AChE (Carlock et al., 1999; Moser et al., 2010).

One of the principal advantages of PBPK modeling to toxicologists is that it enables the estimation of internal concentrations of chemicals/metabolites or biological changes that are not accessible in humans. In this study, the PBPK/PD model was used to predict the target tissue dose (carbaryl concentrations in the brain), which is a better surrogate to adverse effects than are the intake doses. Our analyses suggest that spot 1-N concentrations in urine are better than spot carbaryl concentrations in plasma at predicting 24-h averaged carbaryl concentrations in brain (spot 1-N vs. average brain concentrations: slope = 0.54, *R*^2^ = 0.39; spot plasma vs. average brain concentrations; slope = 0.35, *R*^2^ = 0.29; Figure 6). The common assumption for chemicals with short half-lives is that a blood biomarker is better than a urine biomarker; however, our results indicate that this assumption is not always true. Part of the reason for our observation relates to the nature of “spot” samples, which are collected at a specific moment in time. When a spot urine sample is collected, it contains chemicals accumulated in the bladder, so it is actually time-averaged to some extent. In contrast, a plasma sample is considered a true “snapshot” of the instantaneous concentration of a chemical in blood at a particular time point. Since urinary biomarkers inherently integrate exposures through time, they may be better correlated to biological effects than a spot plasma concentration.

The current study demonstrates the use of computational models to evaluate the utility of various biomarkers. One caveat of the findings in this study is that the best biomarker of exposure, 1-N in urine, and the best biomarker of biochemical changes, RBC AChE inhibition, are both non-specific biomarkers. For 1-N, this compound is a metabolite of both carbaryl and naphthalene, and therefore 1-N found in urine can be a result of exposure to carbaryl, naphthalene, or both. Specificity can be achieved in biomonitoring studies by concurrently measuring 2-naphthol (2-N) concentrations, since 2-N is a metabolite of naphthalene but not of carbaryl (Meeker et al., 2007). Since our model does not take naphthalene exposure into account, our conclusions represent the case in which exposure to naphthalene is negligible. For RBC AChE, this enzyme can be inhibited by several chemicals. The most commonly known inhibitors are the organophosphate pesticides. A test of RBC AChE inhibition cannot distinguish between inhibition due to carbaryl or organophosphates, so again a concurrent measurement of another biomarker, such as 1-N, is necessary to provide some specificity. Of course, from a clinical standpoint, since cholinergic poisonings are all treated similarly it may not be crucial in every case to establish the identity of the toxicant, but from a scientific standpoint it is essential to understand the fundamental limitations of the biomarker approach to estimating exposures and predicting effects based on a single biomarker measurement.

As demonstrated in this study, PBPK/PD models, which are unique in their ability to incorporate a wide variety of research findings, are well-suited for fostering improved use of biomarkers. Computational models provide new avenues for the analysis and interpretation of biomarker data which will contribute to a more detailed understanding of chemical exposures and biochemical effects in human populations. In addition, they can facilitate the discovery of new biomarkers. To realize the full potential of biomonitoring surveys, biomarker data must be combined with any and all available tools to support a fuller understanding of the linkages between exposure, internal dose, and toxicological effect.

### Conflict of interest statement

The authors declare that the research was conducted in the absence of any commercial or financial relationships that could be construed as a potential conflict of interest.
