# Supplementary material for: Analysis of biomarker utility using a PBPK/PD model for carbaryl
Source: Front Pharmacol. 2014 Nov 18;5:246. doi: 10.3389/fphar.2014.00246 (PMC4235294; doi:10.3389/fphar.2014.00246)
Supplement: Supplementary file 1 [file DataSheet1.DOCX]

***Supplementary Material***

**Analysis of biomarker utility using a PBPK model for carbaryl**

**Martin B. Phillips^1^, Miyoung Yoon^2^, Bruce Young^3^, Yu-Mei Tan^4^***

^1^National Exposure Research Laboratory, US Environmental Protection Agency, Duluth, MN, USA

^2^Institute for Chemical Safety Sciences, The Hamner Institutes for Health Sciences, Research Triangle Park, NC, USA

^3^Bayer CropScience, Research Triangle Park, NC, USA

^4^National Exposure Research Laboratory, US Environmental Protection Agency, Research Triangle Park, NC, USA

*** Correspondence:** Dr. Yu-Mei Tan, National Exposure Research Laboratory, US Environmental Protection Agency, 109 TW Alexander Dr, Mail Code E205-01, Research Triangle Park, NC, 27711, USA.

tan.cecilia@epa.gov

**Supplementary Figure 1.** Time course results for a single simulated individual over a 48 hour period. (A) Exposure magnitude (ng carbaryl); (B) concentration of carbaryl in plasma (fg/mL); (C) percent acetylcholinesterase (AChE) inhibition in red blood cells (solid line) and in brain tissue (dotted line); (D) concentration of carbaryl in brain tissue (pM); (E) cumulative amount of 1-naphthol (1-N) eliminated to the bladder compartment (nmol); (F) approximation of spot urine concentration of 1-N if the urine sample had been taken at a particular time point (rate of urinary elimination [nmol/h] divided by rate of urine output [L/h] approximates the spot urine concentration [nmol/L, or nM]).

**Supplementary Figure 2.** Correlations between six model-output variables and the total dose for the 24 hour period prior to the sampling time (ng/kg/day). (A) biomarker of exposure: spot 1-naphthol (1-N) in urine (nM); (B) target tissue concentration: carbaryl concentrations in brain averaged over the 24 hours prior to sampling (pM); (C) biomarker of early biochemical changes at the target tissue: percent acetylcholinesterase (AChE) inhibition in brain tissue (baseline = 0%); (D) biomarker of early biochemical changes at peripheral tissue: percent AChE inhibition in red blood cells (baseline = 0%); (E) percent AChE inhibition in brain tissue averaged over the last 24 hours prior to the urine sampling time; (F) percent AChE inhibition in red blood cells averaged over the last 24 hours prior to the urine sampling time.

**Supplementary Table 1.** Results of sensitivity analysis. The first two columns show which parameters were determined to be sensitive, both a plain-language description (column 1) and the parameter name from the code (column 2). The last five columns indicate which parameters were sensitive for which endpoints. These variables are listed in plain language (row 2) and the name of the endpoint variable in the code is given (row 3). Acronyms: 1-N (1-naphthol), RBCs (red blood cells), AChE (acetylcholinesterase), BW (body weight).

**Supplementary Table 2.** Monte Carlo randomization of sensitive parameters. The first column lists the parameter name (see Supplementary Table 1 for a plain language description). The second column lists whether the parameter is normally or lognormally distributed. The third column gives the central tendency (the mean or geometric mean of the distribution). The fourth column gives the variability (the standard deviation or geometric standard deviation). All distributions were truncated to the central 95% of the distribution to prevent the selection of extreme values.

**MODEL CODE**

Nine m-files were used to generate the data for this manuscript. They are:

carbarylPBPK.m

carbarylPBPK_forSA.m

clip.m

clipCV.m

initCarbaryl_param.m

initCarbaryl_param_MC.m

logclip.m

runScript.m

sensitivityAnalysis.m

The code is included below. The title for the m-files are bolded, centered, and italicized at the top of each file. runScript.m is the file that actually runs the simulations; the others are subordinate to it or were used to conduct the sensitivity analysis.

***carbarylPBPK.m***

function [out] = carbarylPBPK(Exposures,Weight)

% Human PBPK/PD model for Carbaryl supported w/in vitro data

% simplified from Nong et al 2008 (cblfix.model, MCSim)

% revisions ducumented in rat model (carbsimplefree.csl)

% 8/4 based on Knaak 1968 urine data, fa included representing intestinal metabolism/lower bioavailability of carbaryl in human

% by Miyoung Yoon, last revision on August 14, 2011

% The one we want%

% *************** PHYSIOLOGICAL PBPK PARAMETERS ***************

P0 = initCarbaryl_param_MC(Weight);

BW = P0(1); % body weight kg

HCT = P0(2); % Hematocrit fraction

% VOLUMES (fraction of BW) Brown et al., 1997

VBRNC = P0(3); % brain

VFATC = P0(4); % fat

VLIVC = P0(5); % liver

VBLDC = P0(6); % blood

VTBC = P0(7); % fraction of tissue plasma (as multiples of tissue volume)

VBODC = P0(8); % excluding hard bone mass & non-perfused mass such GI content, seminal vesicle content, bladder content

% FLOWS (fraction of QC) Brown et al., 1997

QCC = P0(9); % cardiac output L/h/kg^0.75

QBRNC = P0(10); % brain

QFATC = P0(11); % fat

QLIVC = P0(12); % liver

QBODC = P0(13);

% *************** CARBARYL PBPK PARAMETERS ***************

% Distribution coefficients, tissue:blood (Experimentally determined eq dialysis)

PBRN = P0(14); % brain

PFAT = P0(15); % fat

PBOD = P0(16); % rest of body, muscle value

PLIV = P0(17); % liver

PRBC = P0(18); % RBC:plasma partition

% Fraction unbound in Tissues & blood

FuLiv = P0(19);

FuBrn = P0(20);

FuPls = P0(21);

FuRBC = P0(22);

% Tissue Permeability-area constants (L/h/kg^0.75) Nong et al posteriors

PAFC = P0(23); % fat

PARC = P0(24); % rest of body

PALC = P0(25); % liver

PABC = P0(26); % brain, diffusion limited

PARBC = P0(27);

% Biochemical parameters, from in vitro data

VKM1C = P0(28); % CL for carbaryl to other metabolites (L/hr/kg liver)

Vmax2C = P0(29); % Vmax for carbaryl to 1-naphthol (umol/hr/kg liver)

Km2 = P0(30); % M-M constant for carbaryl to 1-naphthol (uM)

Vmax3C = P0(31); % Vmax for 1-naphthol sulfation (umol/hr/kg liver)

Km3 = P0(32); % uM

Ki3 = P0(33); % uM

Vmax4C = P0(34); % Vmax for 1-naphthol to other 1-napthol metabolites (umol/hr/kg liver)

Km4 = P0(35); % uM

kc = P0(36); % /hr

VKM2C = P0(37); % CL for carbaryl to 1-naphthol in plasma (L/hr/kg plasma)

% GI absorption constants

Ka = P0(38); % /hr, 1st order absorption rate, GI to liver (Houston et al., 1974)

KBileC = P0(39); % first order biliary excretion scalar (/hr/kg^-0.25)

Fa = P0(40); % fraction absorbed to systemic circulation

Fm = P0(41); % fraction of unabsorbed carbaryl absorbed as 1-naphthol

% *************** 1- Compartment for 1-naphthol ***************

% Biochemical

KenBC = P0(42); % urinary elimination of 1- naphthol and its metabolites (/hr/kg^-0.25)

%********* Added parameters for Urinary and Exposure **********

VPEE = P0(43); % Volume of urine void

LAST_VOID = P0(44); % Time of last void in hours

FLOW_RATE = P0(45); % Flow rate

% ************************ SCALING FUNCTIONS ***********************

% VOLUMES (L)

VBRN = VBRNC * BW; % brain

VFAT = VFATC * BW; % fat

VLIV = VLIVC * BW; % liver

VBLD = VBLDC * BW; % blood

VBOD = VBODC * BW; % remaining

VPLS = VBLD *(1-HCT); % plasma

VRBC = VBLD * HCT; % RBC

VBAL = (BW - VBRN - VFAT - VLIV - VBLD - VBOD)/BW-0.15; % assuming 85% perfused volume in the body

% Blood flows (L/hr)

QC = QCC * (BW^0.75) * (1-HCT); % cardiac output (plasma)

QBRN = QBRNC * QC; % plasma flow to brain

QFAT = QFATC * QC; % plasma flow to fat

QLIV = QLIVC * QC; % plasma flow to liver

QBOD = QBODC * QC; % plasma flow to remaining

QBAL = QC - QBRN - QFAT - QLIV - QBOD; % to check blood flow balance

% Permeability to BW^3/4 (diffusional uptake/efflux CL into and out of tissue)

PAF = PAFC * (BW^0.75); % fat

PAR = PARC * (BW^0.75); % remaining

PAL = PALC * (BW^0.75); % liver

PAB = PABC * (BW^0.75); % brain

% Scaling of metabolism and excretion parameters

VKM1 = VKM1C * VLIV;

VMax2 = Vmax2C * VLIV;

VMax3 = Vmax3C * VLIV;

VMax4 = Vmax4C * VLIV;

VKM2 = VKM2C * (VBLD*(1-HCT)); % carbaryl CL in plasma

KBile = KBileC * BW^(-0.25);

KenB = KenBC * BW^(-0.25);

% ************************ PD Cholinesterase constants ************************

% Enz activity (umol/hr/kg tissue)

BRache = P0(46); % Brain AChE activity (umol/hr/kg tissue)

Bache = P0(47); % plasma AChE (umol/hr/kg tissue)

Bbche = P0(48); % plasma BuChE (umol/hr/kg tissue)

RBache = P0(49); % RBC AchE (umol/hr/kg tissue)

% Enz Turnover rates (subst hydrolysis, /hr/active site)

TRce=P0(50); % AChE subst hydrolysis (/hr/active site)

TRbe=P0(51); % BChE subst hydrolysis (/hr/active site)

% Cholinesterase rate constants

KiacheRBC = P0(52); % inhibition (/uM/hr)

KracheRBC = P0(53); % regeneration (/hr)

KiacheBRN = P0(54); % inhibition (/uM/hr)

KracheBRN = P0(55); % regeneration (/hr)

KiachePLS = P0(56); % inhibition (/uM/hr)

KrachePLS = P0(57); % regeneration (/hr)

Kibche = P0(61); % inhibition (/uM/hr)

Krbche = P0(63); % regeneration (/hr)

KdacheRBC = P0(58); % degradation (/hr)

KdacheBrn = P0(59); % degradation (/hr)

KdachePls = P0(60); % degradation (/hr)

Kdbche = P0(62); % degradation (/hr)

% Calculation of enzyme active sites (umole)

ABRache0 = BRache*VBRN/TRce; % Total Brain AChE (umoles)

ABache0 = Bache*VPLS/TRce; % Total plasma AChE (umoles)

ABbche0 = Bbche*VPLS/TRbe; % Total plasma BuChE (umoles)

Arbc0 = RBache*VRBC/TRce; % Total RBC AChE (umoles)

% Calculation of enzyme zero-order synthesis rate constants

Ksache = ABRache0*KdacheBrn; % Brain Syn rate of AChE (umole/hr)

Ksbache = ABache0*KdachePls; % Plasma Syn rate of AChE (umole/hr)

Ksbbche = ABbche0*Kdbche; % Plasma Syn rate of BChE (umole/hr)

Ksrbce = Arbc0*KdacheRBC; % Synthesis rate of RBC (umole/hr)

% ******************** MODEL CONTROLS ********************

% Dosing

IVDOSE = P0(64); % IV dose (ug/kg bw)

MW = 201.2; % mol wt for carbaryl (g/mol)

ODOSE = 0.; % oral dose (ug/kg bw)

TOD = 0;

OD = ODOSE * BW / MW; % oral dose (umoles)

IVD = IVDOSE * BW / MW; % iv dose (umoles)

% Simulation

TSTOP = 168.; % endtime simulation

POINTS = 1000.; % number of outputs

TINF = TSTOP/POINTS; % timed output step ???? This should really be set based on the actual length of time that the IV infusion lasts so that it is indepedent of the length of the simulation and the number of output points

%{

%%%%%%%%%%%%%%%%%%---------!!!!!!!!!!!! This section required major changes when translating to MATLAB, it has been left here FOR REFERENCE ONLY

% ADDED From GDIT Code %%%%%%%%%%%%%%%%%%%%%%%%%%%%%%

% Oral dosing information: dose times, dose amounts

% Use -1 as a dose time to indicate no dose

% Doses are BW scaled (mg/kg)

% BIG = Bolus InGestion

% BIG_AMTS is the vector of a subjects amounts over ndays

% BIG_TIMES is the vector of time (hr) corresponding to subject's amounts over ndays

PARAMETER (MAX_BIG = 142) % 142

DIMENSION BIG_AMTS(MAX_BIG), BIG_TIMES(MAX_BIG)

BIG_AMTS = MAX_BIG*0.0

BIG_TIMES = MAX_BIG*-1.0

AMTDIET = 0. % accumulated amount diet

SAMP_TIME = -1.0 % time of urine sample

TbetweenVoids = 2

% Script for ODOSE

% Schedule the PO dose events

INTEGER BIG_IDX

DO HeyNow BIG_IDX=1,MAX_BIG

IF(BIG_TIMES(BIG_IDX) .ge. 0.0) THEN

schedule BIG_EVENT .at. BIG_TIMES(BIG_IDX)

ENDIF

HeyNow:CONTINUE

% reset the counter for use in the discrete section below

BIG_IDX =1

BIG_TOTAL_DOSE =0

% Added for urine sample

SCHEDULE SAMPLING_EVENT .at. SAMP_TIME

SCHEDULE SAMPLING_EVENT .at. LAST_VOID

END %INITIAL

DYNAMIC

ALGORITHM IALG = 2 % Gear's Stiff

MAXTERVAL MAXT = 1.0e9

MINTERVAL MINT = 1.0e-20

% ************************* DOSING ALGORITHMS *************************

% iv dose

DISCRETE IVdosing

INTERVAL IVint = 24.0

SCHEDULE IVOff .AT. T + TINF

%RIV = IVD/TINF % IV infusion rate (umol/hr)

END

DISCRETE IVOff

%RIV = 0.0

END

% ADDED From GDIT Code %%%%%%%%%%%%%%%%%%%%%%%%%%%%%%

% The accumulated dietary exposure directly attach to the gut compartment with time profile events (Bolus)

DISCRETE BIG_EVENT

%ODOSE= ODOSE + BIG_AMTS(BIG_IDX)*BW/MW

TOD = TOD + BIG_AMTS(BIG_IDX)*BW/MW; % accumulated amount diet

BIG_IDX = BIG_IDX + 1;

BIG_TOTAL_DOSE = BIG_TOTAL_DOSE + BIG_AMTS(BIG_IDX)*BW/MW; %cumulative BIG_AMTS ingested

END

%}

% Construct events list

TSTART = 0;

nExposures = size(Exposures,1);

if IVDOSE ~= 0

nIVevents = ceil(TSTOP/24);

else

nIVevents = 0;

end

nEvents = 2+2*nIVevents+nExposures;

events = zeros(nEvents,2);

index = 1;

events(index,1) = TSTART;

index=index+1;

if IVDOSE ~= 0

for IVcounter = 1:nIVevents

events(index,1) = 12+(IVcounter-1)*24; % The timing of IV infusions wasn't specified, so I put it at noon, once per day. IF THIS CHANGES, THE CONDITION THAT CONTROLS r(31) IN THE INTEGRATION SECTION ALSO NEEDS TO CHANGE

events(index,1) = 12+(IVcounter-1)*24+TINF;

index=index+2;

end

end

if nExposures ~= 0

for j=1:nExposures

events(index,1) = Exposures(j,1);

events(index,2) = Exposures(j,2);

index = index+1;

end

end

events(index,1) = TSTOP;

[Unique,~,ic] = unique(events(:,1));

sortedevents = zeros(max(ic),2);

sortedevents(:,1) = Unique(:,1);

for oldindex=1:length(ic)

for newindex=1:max(ic)

temp = ic(oldindex)-newindex;

if temp == 0

sortedevents(newindex,2) = sortedevents(newindex,2)+events(oldindex,2);

end

end

end

clear events nEvents

events = sortedevents;

nEvents = length(events(:,1));

y0 = zeros(1,32);

y0(1,21) = ABRache0;

y0(1,24) = Arbc0;

y0(1,26) = ABache0;

y0(1,28) = ABbche0;

allTimesT = zeros(TSTOP*10,1);

Y = zeros(TSTOP*10,32);

m=1;

for p=1:nEvents-1

if events(p+1,1) < 120

[T,Results] = ode15s(@DiffEQ,[events(p,1),events(p+1,1)],y0);

else

[T,Results] = ode15s(@DiffEQ,[events(p,1):0.05:events(p+1,1)],y0);

end

for o=1:length(T)

allTimesT(m,1) = T(o);

Y(m,:) = Results(o,:);

m=m+1;

end

y0 = Results(end,:);

if events(p+1,2) ~= 0

y0(1) = y0(1)+events(p+1,2)*BW/MW;

y0(32) = y0(32)+events(p+1,2)*BW/MW;

end

end

cutoffT = find(allTimesT, 1, 'last' );

out.Time = allTimesT(1:cutoffT,:);

Y = Y(1:cutoffT,:);

%}

% ******************* CARBARYL MODEL EQUATIONS (diffusion limited for all tissues) *******************

out.AG = Y(:,1); % amount of carbaryl in stomach & GI (umole) (AG = TOD + integ(RAG, 0))

out.Aabsorbed = Y(:,2); % absorbed as carbaryl

out.AbsorbedNP = Y(:,3);

out.AVL = Y(:,4); % carbaryl in liver blood (umole)

out.AL = Y(:,5); % umole

out.BileCarb = Y(:,6); % amount excreted in bile (umole)

out.nAX = Y(:,7); % amount converted to 1-naphthol (umole)

out.oAX = Y(:,8); % amount converted to other metabolites (umole)- this is treated as cleared out of body

out.AVF = Y(:,9);

out.AF = Y(:,10);

out.AVR = Y(:,11);

out.AR = Y(:,12);

out.AVBR = Y(:,13);

out.ABR = Y(:,14);

out.APLS = Y(:,15); % amount of carbaryl in blood (umole)

out.CPlasma = Y(:,15)/VPLS/1000*MW; % concentration of carbaryl in plasma (ug/mL) -- note strange units

out.AmRBC = Y(:,16);

out.bnAX = Y(:,17); % total 1-naphthol formed (umole)

out.ANPall = Y(:,18); % Amount of all NP in the compartment (umole)

out.UClNPall = Y(:,19); % cumulative amount of all naphthol excreted in urine (umole)

out.ABRI = Y(:,20); % amount of carbaryl bound to brain AchE (umole)

out.ABRache = Y(:,21); % Uninhibited AchE active sites (umole)

out.ABRachei = Y(:,22); % inhibited AchE active sites in brain (umole)

out.ABI = Y(:,23); % amount carbaryl bound to blood ChEs (umole)

out.Arbc = Y(:,24); % amount active sites uninhibited (umole)

out.Arbci = Y(:,25); % inhibited active sites (umole)

out.ABache = Y(:,26);

out.ABachei = Y(:,27);

out.ABbche = Y(:,28);

out.ABbchei = Y(:,29);

out.ATOTC = Y(:,30); % Total chemical conversion

out.IV = Y(:,31);

out.oraldose = Y(:,32);

%%%%%%............... Carbaryl concentration conversions (uM to mg/kg = ppm)..................%%%%%%%%

out.AB = out.APLS+out.AmRBC;

out.PPMLIV=(out.AVL+out.AL)*MW*0.001/VLIV; % carbaryl only radioactivity in liver (ppm)

out.PPMBLD= out.AB*MW*0.001/VBLD; % carbaryl only radioactivity in blood (ppm)

out.PPMFAT=(out.AVF+out.AF)*MW*0.001/VFAT; % carbaryl only radioactivity in fat (ppm)

out.PPMBOD=(out.AVR+out.AR)*MW*0.001/VBOD; % carbaryl only radioactivity in rest of body (ppm)

out.PPMBRN=(out.ABR+out.AVBR)*MW*0.001/VBRN; % carbaryl only radioactivity in brain (ppm)

out.PPMPLS=out.APLS*MW*0.001/VPLS; % carbaryl only radioactivity in plasma (ppm)

out.PPMRBC=out.AmRBC*MW*0.001/VRBC; % carbaryl only radioactivity in red cells (ppm)

out.PBRache = 100*(out.ABRache/ABRache0); % remaining activity compared to control

out.PBRNdep = 100-out.PBRache; % depression compared to control

%%%___Blood___%%%

% Plasma

out.PBLTche = 100*((out.ABache+out.ABbche)/(ABache0+ABbche0)); % Total remaining plasma ChEs activity

out.PBLache = 100*(out.ABache/ABache0); % Remaining plasma AChE activity

out.PBLbche = 100*(out.ABbche/ABbche0); % Remaining plasma BChE activity

out.PBLDdep = 100-out.PBLTche; % Total plasma ChEs depression (%) vs control

out.PPLSAdep = 100-out.PBLache; % Plasma AchE depression (%)

% RBC

out.PRBCche = 100*(out.Arbc/Arbc0); % Remaining RBC AChE activity

out.PRBCdep = 100-out.PRBCche; % Remaining RBC AChE depression (%) vs control

% Whole blood

out.PWBTche = 100*(out.ABache+out.ABbche+out.Arbc)/(ABache0+ABbche0+Arbc0); % Total remaining blood ChEs

out.PWBache = 100*(out.ABache+out.Arbc)/(ABache0+Arbc0); % Total remaining blood AChE activity

out.PWBbche = out.PBLbche; % Total remaining blood BChE activity ????

out.PBLTdep = 100-out.PWBTche; % Total blood ChEs depression (%)

% *************** Mass Balance ***************%

% Total dose (umol)

% out.TotDose = OD + out.IV + BIG_TOTAL_DOSE; old equation from acslX, replaced with next line

out.TotDose = out.IV + out.oraldose;

out.TDOSE = out.Aabsorbed + out.AbsorbedNP + out.IV;

% Cumulative Amount eliminated in urine as 1-naphthol derivatives

for nTime = 1:cutoffT

if out.TotDose(nTime,1) ~= 0

out.PUCL(nTime,1) = (out.UClNPall(nTime,1)/out.TotDose(nTime,1))*100;

else

out.PUCL(nTime,1) = 0;

end

end

% Amount in body (umol)

out.TMASSc = out.AL + out.AVL + out.AF + out.AVF + out.AR + out.AVR + out.ABR + out.AVBR + out.APLS + out.AmRBC;

out.TMASSn = out.ANPall;

% Amount present as bound to ChEs (umole)

out.TMASSi = out.ABRachei + out.Arbci + out.ABachei + out.ABbchei;

% Amount eliminated

out.TMASSEL = out.oAX+out.BileCarb+out.UClNPall;

% Mass balance equations (%)

out.TOTBAL = (out.TDOSE - out.TMASSEL - out.TMASSi - out.TMASSc - out.TMASSn)./(out.TotDose+1e-10); % fraction of absorbed dose

%**************** Urine Sample ************

out.lNconcl = out.UClNPall/VPEE; % sensitivity = 1Nconc1(SAMP_TIME) - 1Nconc1(LAST_VOID), I think

out.lNconcm = KenB*out.ANPall/FLOW_RATE; % sensitivity at SAMP_TIME

function dy = DiffEQ(TIME,y)

AG = y(1);

AVL = y(4);

AL = y(5);

oAX = y(8);

AVF = y(9);

AF = y(10);

AVR = y(11);

AR = y(12);

AVBR = y(13);

ABR = y(14);

APLS = y(15);

AmRBC = y(16);

ANPall = y(18);

UClNPall = y(19);

ABRache = y(21);

ABRachei = y(22);

Arbc = y(24);

Arbci = y(25);

ABache = y(26);

ABachei = y(27);

ABbche = y(28);

ABbchei = y(29);

CVL = y(4)/(VTBC*VLIV); % umole/L

CL = y(5)/((1-VTBC)*VLIV); % umole/L

% Carbaryl metabolism in liver (only oxidative metabolism in human liver based on in vitro data)

CVF = y(9)/(VTBC*VFAT);

CF = y(10)/((1-VTBC)*VFAT);

CVR = y(11)/(VTBC*VBOD);

CR = y(12)/((1-VTBC)*VBOD);

CVBR = y(13)/(VTBC*VBRN);

CBR = y(14)/((1-VTBC)*VBRN);

CPLS = y(15)/VPLS; % blood concentration (umole/L)

CV = (QBRN*CVBR+QBOD*CVR+QFAT*CVF+QLIV*CVL)/QC; % mixed venous blood concentration (umole/L)

CRBC = y(16)/VRBC;

PlsAB = y(15); % carbaryl Amnt in Plasma (umole) ????

RBCAB = y(16); % carbaryl Amnt in RBC (umole) ????

% ********************** END OF CARBARYL EQUATIONS ***************

%%%%%%%%%%%%%%%%%%%%%%%%%%%%%%%%%%%%%%%%%%%%%%%%%%%%%%%%%%%%%%%%%%%%%%%%%%%%%%%%%%%%%%%%%%%%%%%%%%%%%%%%%%%%%%%

% ************ 1 Compartment for the sum of free 1-NAPHTHOL and its metabolites ************

CLN = y(18)/VPLS;

%%%%% ************** PharmacoDynamic model for cholinesterase interaction *************************

%%%___BRAIN____%%%

% Calculating Brain AchE depression (%)

r = zeros(32,1);

r(1) = -Ka*AG; % rAG: rate of carbaryl absorption in stomach & GI (umole/hr)

r(2) = Fa*Ka*AG*(1-Fm); % rAbsorbed

r(3) = Fa*Ka*AG*Fm; % rAbsorbedNP: absorbed as 1-naphthol

r(4) = QLIV*(CPLS - CVL) + Fa*Ka*AG*(1-Fm) - kc*AVL +PAL*(CL/PLIV - CVL); % rAVL: liver tissue blood (umole/hr)

r(6) = KBile* VLIV *(CL*FuLiv); % rBile: liver biliary excretion of free carbaryl (umole/hr)

r(7) = VMax2* (CL*FuLiv)/(Km2+(CL*FuLiv)); % nRAM: rate of metabolism to 1-naphthol (umole/hr)

r(8) = VKM1 * (CL*FuLiv); % oRAM: rate of metabolism to other metabolites (umole/hr)

r(5) = PAL*(CVL-CL/PLIV) - kc*AL - r(6) - r(7) - r(8); % rAL: liver (umole/hr)

r(9) = QFAT*(CPLS - CVF) -kc*AVF + PAF*(CF/PFAT - CVF); % rAVF: fat tissue blood (umole/hr)

r(10) = PAF*(CVF-CF/PFAT) - kc*AF; % rAF

r(11) = QBOD*(CPLS - CVR) - kc*AVR + PAR*(CR/PBOD-CVR); % rAVR: remaining tissue blood (umole/hr)

r(12) = PAR*(CVR-CR/PBOD) - kc*AR; % rAR

r(13) = QBRN*(CPLS - CVBR) + PAB*(CBR/PBRN - CVBR)-kc*AVBR; % rAVBR: brain tissue blood (umole/hr)

r(17) = VKM2*CPLS*FuPls; % bnRAM: plasma rate of metabolism to 1-naphthol (umole/hr)

r(19) = KenB * ANPall; % nKeB: Rate of naphthol elimination into urine (umole/hr)

r(20) = KiacheBRN * ABRache *(CBR*FuBrn); % rABRI: rate of carbaryl binding to brain AchE (umole/hr; bimolecular reaction)

r(14) = PAB*(CVBR - CBR/PBRN) - kc*ABR - r(20); % rABR: brain tissue diffussion w/ Che interaction (umole/hr)

r(21) = Ksache - ABRache*KdacheBrn - KiacheBRN*ABRache*(CBR*FuBrn) + ABRachei*KracheBRN; % rABRache: rate of change in brain AchE with carbaryl interaction (umole/hr)

r(22) = KiacheBRN*ABRache*(CBR*FuBrn)-ABRachei*KracheBRN; % rABRachei: rate of change in inhibited AchE active sites (umole/hr)

r(23) = KiachePLS*ABache*CPLS*FuPls+Kibche*ABbche*CPLS*FuPls+KiacheRBC*Arbc*CRBC*FuRBC; % rABI: rate of carbaryl binding to blood ChEs (umole/hr; bimolecular reaction)

r(18) = kc*(AL+AVL+AF+AVF+AR+AVR+AVBR+ABR+APLS+AmRBC)+r(7)+r(20)+r(23)+r(17)-r(19)+r(3); % rANPall: rate of NP ALL in the compartment (umole/hr)

r(24) = Ksrbce - KdacheRBC*Arbc - KiacheRBC*Arbc*CRBC*FuRBC + Arbci*KracheRBC; % rArbc: rate of RBC AchE active sties (umole/hr)

r(25) = KiacheRBC*Arbc*CRBC*FuRBC - Arbci*KracheRBC; % rArbci: rate of inhibited active sites (umole/hr)

r(16) = PARBC*(CPLS-CRBC/PRBC)-r(25)-kc*AmRBC; % rAmRBC

r(26) = Ksbache-ABache*KdachePls-KiachePLS*ABache*CPLS*FuPls + ABachei*KrachePLS; % rABache

r(27) = KiachePLS*ABache*CPLS*FuPls - ABachei*KrachePLS; % rABachei

r(28) = Ksbbche - ABbche*Kdbche - Kibche*ABbche*CPLS*FuPls + ABbchei*Krbche; % rABbche: rate bche

r(29) = Kibche*ABbche*CPLS*FuPls - ABbchei*Krbche; % rABbchei: bche inhibition, error corrected ABbchei*Krache->*Krbche

r(30) = kc*(AL+AVL+AF+AVF+AR+AVR+ABR+AVBR+APLS+AmRBC); % rTOTC

if mod(TIME,24) >= 12 && mod(TIME,24) < 12+TINF

r(31) = IVD/TINF; % rIV: IV infusion rate (umol/hr)

else

r(31) = 0;

end

r(15) = QC*(CV-CPLS)-r(27)-r(29)-r(17)-kc*APLS+r(31)-PARBC*(CPLS-CRBC/PRBC); % rAPLS: rate of carbaryl change in blood including iv dosing (umole/hr)

r(32) = 0;

dy = r;

end

end

***carbarylPBPK_forSA.m***

function [out] = carbarylPBPK_forSA(Exposures,Weight,P0) %XXXX added P0 to end of list

% Human PBPK/PD model for Carbaryl supported w/in vitro data

% simplified from Nong et al 2008 (cblfix.model, MCSim)

% revisions ducumented in rat model (carbsimplefree.csl)

% 8/4 based on Knaak 1968 urine data, fa included representing intestinal metabolism/lower bioavailability of carbaryl in human

% by Miyoung Yoon, last revision on August 14, 2011

% The one we want%

% *************** PHYSIOLOGICAL PBPK PARAMETERS ***************

%P0 = initCarbaryl_param(Weight); %XXXX commented this out

BW = P0(1); % body weight kg

HCT = P0(2); % Hematocrit fraction

% VOLUMES (fraction of BW) Brown et al., 1997

VBRNC = P0(3); % brain

VFATC = P0(4); % fat

VLIVC = P0(5); % liver

VBLDC = P0(6); % blood

VTBC = P0(7); % fraction of tissue plasma (as multiples of tissue volume)

VBODC = P0(8); % excluding hard bone mass & non-perfused mass such GI content, seminal vesicle content, bladder content

% FLOWS (fraction of QC) Brown et al., 1997

QCC = P0(9); % cardiac output L/h/kg^0.75

QBRNC = P0(10); % brain

QFATC = P0(11); % fat

QLIVC = P0(12); % liver

QBODC = P0(13);

% *************** CARBARYL PBPK PARAMETERS ***************

% Distribution coefficients, tissue:blood (Experimentally determined eq dialysis)

PBRN = P0(14); % brain

PFAT = P0(15); % fat

PBOD = P0(16); % rest of body, muscle value

PLIV = P0(17); % liver

PRBC = P0(18); % RBC:plasma partition

% Fraction unbound in Tissues & blood

FuLiv = P0(19);

FuBrn = P0(20);

FuPls = P0(21);

FuRBC = P0(22);

% Tissue Permeability-area constants (L/h/kg^0.75) Nong et al posteriors

PAFC = P0(23); % fat

PARC = P0(24); % rest of body

PALC = P0(25); % liver

PABC = P0(26); % brain, diffusion limited

PARBC = P0(27);

% Biochemical parameters, from in vitro data

VKM1C = P0(28); % CL for carbaryl to other metabolites (L/hr/kg liver)

Vmax2C = P0(29); % Vmax for carbaryl to 1-naphthol (umol/hr/kg liver)

Km2 = P0(30); % M-M constant for carbaryl to 1-naphthol (uM)

Vmax3C = P0(31); % Vmax for 1-naphthol sulfation (umol/hr/kg liver)

Km3 = P0(32); % uM

Ki3 = P0(33); % uM

Vmax4C = P0(34); % Vmax for 1-naphthol to other 1-napthol metabolites (umol/hr/kg liver)

Km4 = P0(35); % uM

kc = P0(36); % /hr

VKM2C = P0(37); % CL for carbaryl to 1-naphthol in plasma (L/hr/kg plasma)

% GI absorption constants

Ka = P0(38); % /hr, 1st order absorption rate, GI to liver (Houston et al., 1974)

KBileC = P0(39); % first order biliary excretion scalar (/hr/kg^-0.25)

Fa = P0(40); % fraction absorbed to systemic circulation

Fm = P0(41); % fraction of unabsorbed carbaryl absorbed as 1-naphthol

% *************** 1- Compartment for 1-naphthol ***************

% Biochemical

KenBC = P0(42); % urinary elimination of 1- naphthol and its metabolites (/hr/kg^-0.25)

%********* Added parameters for Urinary and Exposure **********

VPEE = P0(43); % Volume of urine void

LAST_VOID = P0(44); % Time of last void in hours

FLOW_RATE = P0(45); % Flow rate

% ************************ SCALING FUNCTIONS ***********************

% VOLUMES (L)

VBRN = VBRNC * BW; % brain

VFAT = VFATC * BW; % fat

VLIV = VLIVC * BW; % liver

VBLD = VBLDC * BW; % blood

VBOD = VBODC * BW; % remaining

VPLS = VBLD *(1-HCT); % plasma

VRBC = VBLD * HCT; % RBC

VBAL = (BW - VBRN - VFAT - VLIV - VBLD - VBOD)/BW-0.15; % assuming 85% perfused volume in the body

% Blood flows (L/hr)

QC = QCC * (BW^0.75) * (1-HCT); % cardiac output (plasma)

QBRN = QBRNC * QC; % plasma flow to brain

QFAT = QFATC * QC; % plasma flow to fat

QLIV = QLIVC * QC; % plasma flow to liver

QBOD = QBODC * QC; % plasma flow to remaining

QBAL = QC - QBRN - QFAT - QLIV - QBOD; % to check blood flow balance

% Permeability to BW^3/4 (diffusional uptake/efflux CL into and out of tissue)

PAF = PAFC * (BW^0.75); % fat

PAR = PARC * (BW^0.75); % remaining

PAL = PALC * (BW^0.75); % liver

PAB = PABC * (BW^0.75); % brain

% Scaling of metabolism and excretion parameters

VKM1 = VKM1C * VLIV;

VMax2 = Vmax2C * VLIV;

VMax3 = Vmax3C * VLIV;

VMax4 = Vmax4C * VLIV;

VKM2 = VKM2C * (VBLD*(1-HCT)); % carbaryl CL in plasma

KBile = KBileC * BW^(-0.25);

KenB = KenBC * BW^(-0.25);

% ************************ PD Cholinesterase constants ************************

% Enz activity (umol/hr/kg tissue)

BRache = P0(46); % Brain AChE activity (umol/hr/kg tissue)

Bache = P0(47); % plasma AChE (umol/hr/kg tissue)

Bbche = P0(48); % plasma BuChE (umol/hr/kg tissue)

RBache = P0(49); % RBC AchE (umol/hr/kg tissue)

% Enz Turnover rates (subst hydrolysis, /hr/active site)

TRce=P0(50); % AChE subst hydrolysis (/hr/active site)

TRbe=P0(51); % BChE subst hydrolysis (/hr/active site)

% Cholinesterase rate constants

KiacheRBC = P0(52); % inhibition (/uM/hr)

KracheRBC = P0(53); % regeneration (/hr)

KiacheBRN = P0(54); % inhibition (/uM/hr)

KracheBRN = P0(55); % regeneration (/hr)

KiachePLS = P0(56); % inhibition (/uM/hr)

KrachePLS = P0(57); % regeneration (/hr)

Kibche = P0(61); % inhibition (/uM/hr)

Krbche = P0(63); % regeneration (/hr)

KdacheRBC = P0(58); % degradation (/hr)

KdacheBrn = P0(59); % degradation (/hr)

KdachePls = P0(60); % degradation (/hr)

Kdbche = P0(62); % degradation (/hr)

% Calculation of enzyme active sites (umole)

ABRache0 = BRache*VBRN/TRce; % Total Brain AChE (umoles)

ABache0 = Bache*VPLS/TRce; % Total plasma AChE (umoles)

ABbche0 = Bbche*VPLS/TRbe; % Total plasma BuChE (umoles)

Arbc0 = RBache*VRBC/TRce; % Total RBC AChE (umoles)

% Calculation of enzyme zero-order synthesis rate constants

Ksache = ABRache0*KdacheBrn; % Brain Syn rate of AChE (umole/hr)

Ksbache = ABache0*KdachePls; % Plasma Syn rate of AChE (umole/hr)

Ksbbche = ABbche0*Kdbche; % Plasma Syn rate of BChE (umole/hr)

Ksrbce = Arbc0*KdacheRBC; % Synthesis rate of RBC (umole/hr)

% ******************** MODEL CONTROLS ********************

% Dosing

IVDOSE = P0(64); % IV dose (ug/kg bw)

MW = 201.2; % mol wt for carbaryl (g/mol)

ODOSE = 0.; % oral dose (ug/kg bw)

TOD = 0;

OD = ODOSE * BW / MW; % oral dose (umoles)

IVD = IVDOSE * BW / MW; % iv dose (umoles)

% Simulation

TSTOP = 168.; % endtime simulation

POINTS = 1000.; % number of outputs

TINF = TSTOP/POINTS; % timed output step ???? This should really be set based on the actual length of time that the IV infusion lasts so that it is indepedent of the length of the simulation and the number of output points

%{

%%%%%%%%%%%%%%%%%%---------!!!!!!!!!!!! This section required major changes when translating to MATLAB, it has been left here FOR REFERENCE ONLY

% ADDED From GDIT Code %%%%%%%%%%%%%%%%%%%%%%%%%%%%%%

% Oral dosing information: dose times, dose amounts

% Use -1 as a dose time to indicate no dose

% Doses are BW scaled (mg/kg)

% BIG = Bolus InGestion

% BIG_AMTS is the vector of a subjects amounts over ndays

% BIG_TIMES is the vector of time (hr) corresponding to subject's amounts over ndays

PARAMETER (MAX_BIG = 142) % 142

DIMENSION BIG_AMTS(MAX_BIG), BIG_TIMES(MAX_BIG)

BIG_AMTS = MAX_BIG*0.0

BIG_TIMES = MAX_BIG*-1.0

AMTDIET = 0. % accumulated amount diet

SAMP_TIME = -1.0 % time of urine sample

TbetweenVoids = 2

% Script for ODOSE

% Schedule the PO dose events

INTEGER BIG_IDX

DO HeyNow BIG_IDX=1,MAX_BIG

IF(BIG_TIMES(BIG_IDX) .ge. 0.0) THEN

schedule BIG_EVENT .at. BIG_TIMES(BIG_IDX)

ENDIF

HeyNow:CONTINUE

% reset the counter for use in the discrete section below

BIG_IDX =1

BIG_TOTAL_DOSE =0

% Added for urine sample

SCHEDULE SAMPLING_EVENT .at. SAMP_TIME

SCHEDULE SAMPLING_EVENT .at. LAST_VOID

END %INITIAL

DYNAMIC

ALGORITHM IALG = 2 % Gear's Stiff

MAXTERVAL MAXT = 1.0e9

MINTERVAL MINT = 1.0e-20

% ************************* DOSING ALGORITHMS *************************

% iv dose

DISCRETE IVdosing

INTERVAL IVint = 24.0

SCHEDULE IVOff .AT. T + TINF

%RIV = IVD/TINF % IV infusion rate (umol/hr)

END

DISCRETE IVOff

%RIV = 0.0

END

% ADDED From GDIT Code %%%%%%%%%%%%%%%%%%%%%%%%%%%%%%

% The accumulated dietary exposure directly attach to the gut compartment with time profile events (Bolus)

DISCRETE BIG_EVENT

%ODOSE= ODOSE + BIG_AMTS(BIG_IDX)*BW/MW

TOD = TOD + BIG_AMTS(BIG_IDX)*BW/MW; % accumulated amount diet

BIG_IDX = BIG_IDX + 1;

BIG_TOTAL_DOSE = BIG_TOTAL_DOSE + BIG_AMTS(BIG_IDX)*BW/MW; %cumulative BIG_AMTS ingested

END

%}

% Construct events list

TSTART = 0;

nExposures = size(Exposures,1);

if IVDOSE ~= 0

nIVevents = ceil(TSTOP/24);

else

nIVevents = 0;

end

nEvents = 2+2*nIVevents+nExposures;

events = zeros(nEvents,2);

index = 1;

events(index,1) = TSTART;

index=index+1;

if IVDOSE ~= 0

for IVcounter = 1:nIVevents

events(index,1) = 12+(IVcounter-1)*24; % The timing of IV infusions wasn't specified, so I put it at noon, once per day. IF THIS CHANGES, THE CONDITION THAT CONTROLS r(31) IN THE INTEGRATION SECTION ALSO NEEDS TO CHANGE

events(index,1) = 12+(IVcounter-1)*24+TINF;

index=index+2;

end

end

if nExposures ~= 0

for j=1:nExposures

events(index,1) = Exposures(j,1);

events(index,2) = Exposures(j,2);

index = index+1;

end

end

events(index,1) = TSTOP;

[Unique,~,ic] = unique(events(:,1));

sortedevents = zeros(max(ic),2);

sortedevents(:,1) = Unique(:,1);

for oldindex=1:length(ic)

for newindex=1:max(ic)

temp = ic(oldindex)-newindex;

if temp == 0

sortedevents(newindex,2) = sortedevents(newindex,2)+events(oldindex,2);

end

end

end

clear events nEvents

events = sortedevents;

nEvents = length(events(:,1));

y0 = zeros(1,32);

y0(1,21) = ABRache0;

y0(1,24) = Arbc0;

y0(1,26) = ABache0;

y0(1,28) = ABbche0;

allTimesT = zeros(TSTOP*10,1);

Y = zeros(TSTOP*10,32);

m=1;

for p=1:nEvents-1

if events(p+1,1) < 168 %XXXX Changed to 168 from 120

[T,Results] = ode15s(@DiffEQ,[events(p,1),events(p+1,1)],y0);

else

[T,Results] = ode15s(@DiffEQ,[events(p,1):0.05:events(p+1,1)],y0);

end

for o=1:length(T)

allTimesT(m,1) = T(o);

Y(m,:) = Results(o,:);

m=m+1;

end

y0 = Results(end,:);

if events(p+1,2) ~= 0

y0(1) = y0(1)+events(p+1,2)*BW/MW;

y0(32) = y0(32)+events(p+1,2)*BW/MW;

end

end

cutoffT = find(allTimesT, 1, 'last' );

out.Time = allTimesT(1:cutoffT,:);

Y = Y(1:cutoffT,:);

%}

% ******************* CARBARYL MODEL EQUATIONS (diffusion limited for all tissues) *******************

out.AG = Y(:,1); % amount of carbaryl in stomach & GI (umole) (AG = TOD + integ(RAG, 0))

out.Aabsorbed = Y(:,2); % absorbed as carbaryl

out.AbsorbedNP = Y(:,3);

out.AVL = Y(:,4); % carbaryl in liver blood (umole)

out.AL = Y(:,5); % umole

out.BileCarb = Y(:,6); % amount excreted in bile (umole)

out.nAX = Y(:,7); % amount converted to 1-naphthol (umole)

out.oAX = Y(:,8); % amount converted to other metabolites (umole)- this is treated as cleared out of body

out.AVF = Y(:,9);

out.AF = Y(:,10);

out.AVR = Y(:,11);

out.AR = Y(:,12);

out.AVBR = Y(:,13);

out.ABR = Y(:,14);

out.APLS = Y(:,15); % amount of carbaryl in blood (umole)

out.CPlasma = Y(:,15)/VPLS/1000*MW; % concentration of carbaryl in plasma (ug/mL) -- note strange units

out.AmRBC = Y(:,16);

out.bnAX = Y(:,17); % total 1-naphthol formed (umole)

out.ANPall = Y(:,18); % Amount of all NP in the compartment (umole)

out.UClNPall = Y(:,19); % cumulative amount of all naphthol excreted in urine (umole)

out.ABRI = Y(:,20); % amount of carbaryl bound to brain AchE (umole)

out.ABRache = Y(:,21); % Uninhibited AchE active sites (umole)

out.ABRachei = Y(:,22); % inhibited AchE active sites in brain (umole)

out.ABI = Y(:,23); % amount carbaryl bound to blood ChEs (umole)

out.Arbc = Y(:,24); % amount active sites uninhibited (umole)

out.Arbci = Y(:,25); % inhibited active sites (umole)

out.ABache = Y(:,26);

out.ABachei = Y(:,27);

out.ABbche = Y(:,28);

out.ABbchei = Y(:,29);

out.ATOTC = Y(:,30); % Total chemical conversion

out.IV = Y(:,31);

out.oraldose = Y(:,32);

%%%%%%............... Carbaryl concentration conversions (uM to mg/kg = ppm)..................%%%%%%%%

out.AB = out.APLS+out.AmRBC;

out.PPMLIV=(out.AVL+out.AL)*MW*0.001/VLIV; % carbaryl only radioactivity in liver (ppm)

out.PPMBLD= out.AB*MW*0.001/VBLD; % carbaryl only radioactivity in blood (ppm)

out.PPMFAT=(out.AVF+out.AF)*MW*0.001/VFAT; % carbaryl only radioactivity in fat (ppm)

out.PPMBOD=(out.AVR+out.AR)*MW*0.001/VBOD; % carbaryl only radioactivity in rest of body (ppm)

out.PPMBRN=(out.ABR+out.AVBR)*MW*0.001/VBRN; % carbaryl only radioactivity in brain (ppm)

out.PPMPLS=out.APLS*MW*0.001/VPLS; % carbaryl only radioactivity in plasma (ppm)

out.PPMRBC=out.AmRBC*MW*0.001/VRBC; % carbaryl only radioactivity in red cells (ppm)

out.PBRache = 100*(out.ABRache/ABRache0); % remaining activity compared to control

out.PBRNdep = 100-out.PBRache; % depression compared to control

%%%___Blood___%%%

% Plasma

out.PBLTche = 100*((out.ABache+out.ABbche)/(ABache0+ABbche0)); % Total remaining plasma ChEs activity

out.PBLache = 100*(out.ABache/ABache0); % Remaining plasma AChE activity

out.PBLbche = 100*(out.ABbche/ABbche0); % Remaining plasma BChE activity

out.PBLDdep = 100-out.PBLTche; % Total plasma ChEs depression (%) vs control

out.PPLSAdep = 100-out.PBLache; % Plasma AchE depression (%)

% RBC

out.PRBCche = 100*(out.Arbc/Arbc0); % Remaining RBC AChE activity

out.PRBCdep = 100-out.PRBCche; % Remaining RBC AChE depression (%) vs control

% Whole blood

out.PWBTche = 100*(out.ABache+out.ABbche+out.Arbc)/(ABache0+ABbche0+Arbc0); % Total remaining blood ChEs

out.PWBache = 100*(out.ABache+out.Arbc)/(ABache0+Arbc0); % Total remaining blood AChE activity

out.PWBbche = out.PBLbche; % Total remaining blood BChE activity ????

out.PBLTdep = 100-out.PWBTche; % Total blood ChEs depression (%)

% *************** Mass Balance ***************%

% Total dose (umol)

% out.TotDose = OD + out.IV + BIG_TOTAL_DOSE; old equation from acslX, replaced with next line

out.TotDose = out.IV + out.oraldose;

out.TDOSE = out.Aabsorbed + out.AbsorbedNP + out.IV;

% Cumulative Amount eliminated in urine as 1-naphthol derivatives

for nTime = 1:cutoffT

if out.TotDose(nTime,1) ~= 0

out.PUCL(nTime,1) = (out.UClNPall(nTime,1)/out.TotDose(nTime,1))*100;

else

out.PUCL(nTime,1) = 0;

end

end

% Amount in body (umol)

out.TMASSc = out.AL + out.AVL + out.AF + out.AVF + out.AR + out.AVR + out.ABR + out.AVBR + out.APLS + out.AmRBC;

out.TMASSn = out.ANPall;

% Amount present as bound to ChEs (umole)

out.TMASSi = out.ABRachei + out.Arbci + out.ABachei + out.ABbchei;

% Amount eliminated

out.TMASSEL = out.oAX+out.BileCarb+out.UClNPall;

% Mass balance equations (%)

out.TOTBAL = (out.TDOSE - out.TMASSEL - out.TMASSi - out.TMASSc - out.TMASSn)./(out.TotDose+1e-10); % fraction of absorbed dose

%**************** Urine Sample ************

out.lNconcl = out.UClNPall/VPEE; % sensitivity = 1Nconc1(SAMP_TIME) - 1Nconc1(LAST_VOID), I think

out.lNconcm = KenB*out.ANPall/FLOW_RATE; % sensitivity at SAMP_TIME

function dy = DiffEQ(TIME,y)

AG = y(1);

AVL = y(4);

AL = y(5);

oAX = y(8);

AVF = y(9);

AF = y(10);

AVR = y(11);

AR = y(12);

AVBR = y(13);

ABR = y(14);

APLS = y(15);

AmRBC = y(16);

ANPall = y(18);

UClNPall = y(19);

ABRache = y(21);

ABRachei = y(22);

Arbc = y(24);

Arbci = y(25);

ABache = y(26);

ABachei = y(27);

ABbche = y(28);

ABbchei = y(29);

CVL = y(4)/(VTBC*VLIV); % umole/L

CL = y(5)/((1-VTBC)*VLIV); % umole/L

% Carbaryl metabolism in liver (only oxidative metabolism in human liver based on in vitro data)

CVF = y(9)/(VTBC*VFAT);

CF = y(10)/((1-VTBC)*VFAT);

CVR = y(11)/(VTBC*VBOD);

CR = y(12)/((1-VTBC)*VBOD);

CVBR = y(13)/(VTBC*VBRN);

CBR = y(14)/((1-VTBC)*VBRN);

CPLS = y(15)/VPLS; % blood concentration (umole/L)

CV = (QBRN*CVBR+QBOD*CVR+QFAT*CVF+QLIV*CVL)/QC; % mixed venous blood concentration (umole/L)

CRBC = y(16)/VRBC;

PlsAB = y(15); % carbaryl Amnt in Plasma (umole) ????

RBCAB = y(16); % carbaryl Amnt in RBC (umole) ????

% ********************** END OF CARBARYL EQUATIONS ***************

%%%%%%%%%%%%%%%%%%%%%%%%%%%%%%%%%%%%%%%%%%%%%%%%%%%%%%%%%%%%%%%%%%%%%%%%%%%%%%%%%%%%%%%%%%%%%%%%%%%%%%%%%%%%%%%

% ************ 1 Compartment for the sum of free 1-NAPHTHOL and its metabolites ************

CLN = y(18)/VPLS;

%%%%% ************** PharmacoDynamic model for cholinesterase interaction *************************

%%%___BRAIN____%%%

% Calculating Brain AchE depression (%)

r = zeros(32,1);

r(1) = -Ka*AG; % rAG: rate of carbaryl absorption in stomach & GI (umole/hr)

r(2) = Fa*Ka*AG*(1-Fm); % rAbsorbed

r(3) = Fa*Ka*AG*Fm; % rAbsorbedNP: absorbed as 1-naphthol

r(4) = QLIV*(CPLS - CVL) + Fa*Ka*AG*(1-Fm) - kc*AVL +PAL*(CL/PLIV - CVL); % rAVL: liver tissue blood (umole/hr)

r(6) = KBile* VLIV *(CL*FuLiv); % rBile: liver biliary excretion of free carbaryl (umole/hr)

r(7) = VMax2* (CL*FuLiv)/(Km2+(CL*FuLiv)); % nRAM: rate of metabolism to 1-naphthol (umole/hr)

r(8) = VKM1 * (CL*FuLiv); % oRAM: rate of metabolism to other metabolites (umole/hr)

r(5) = PAL*(CVL-CL/PLIV) - kc*AL - r(6) - r(7) - r(8); % rAL: liver (umole/hr)

r(9) = QFAT*(CPLS - CVF) -kc*AVF + PAF*(CF/PFAT - CVF); % rAVF: fat tissue blood (umole/hr)

r(10) = PAF*(CVF-CF/PFAT) - kc*AF; % rAF

r(11) = QBOD*(CPLS - CVR) - kc*AVR + PAR*(CR/PBOD-CVR); % rAVR: remaining tissue blood (umole/hr)

r(12) = PAR*(CVR-CR/PBOD) - kc*AR; % rAR

r(13) = QBRN*(CPLS - CVBR) + PAB*(CBR/PBRN - CVBR)-kc*AVBR; % rAVBR: brain tissue blood (umole/hr)

r(17) = VKM2*CPLS*FuPls; % bnRAM: plasma rate of metabolism to 1-naphthol (umole/hr)

r(19) = KenB * ANPall; % nKeB: Rate of naphthol elimination into urine (umole/hr)

r(20) = KiacheBRN * ABRache *(CBR*FuBrn); % rABRI: rate of carbaryl binding to brain AchE (umole/hr; bimolecular reaction)

r(14) = PAB*(CVBR - CBR/PBRN) - kc*ABR - r(20); % rABR: brain tissue diffussion w/ Che interaction (umole/hr)

r(21) = Ksache - ABRache*KdacheBrn - KiacheBRN*ABRache*(CBR*FuBrn) + ABRachei*KracheBRN; % rABRache: rate of change in brain AchE with carbaryl interaction (umole/hr)

r(22) = KiacheBRN*ABRache*(CBR*FuBrn)-ABRachei*KracheBRN; % rABRachei: rate of change in inhibited AchE active sites (umole/hr)

r(23) = KiachePLS*ABache*CPLS*FuPls+Kibche*ABbche*CPLS*FuPls+KiacheRBC*Arbc*CRBC*FuRBC; % rABI: rate of carbaryl binding to blood ChEs (umole/hr; bimolecular reaction)

r(18) = kc*(AL+AVL+AF+AVF+AR+AVR+AVBR+ABR+APLS+AmRBC)+r(7)+r(20)+r(23)+r(17)-r(19)+r(3); % rANPall: rate of NP ALL in the compartment (umole/hr)

r(24) = Ksrbce - KdacheRBC*Arbc - KiacheRBC*Arbc*CRBC*FuRBC + Arbci*KracheRBC; % rArbc: rate of RBC AchE active sties (umole/hr)

r(25) = KiacheRBC*Arbc*CRBC*FuRBC - Arbci*KracheRBC; % rArbci: rate of inhibited active sites (umole/hr)

r(16) = PARBC*(CPLS-CRBC/PRBC)-r(25)-kc*AmRBC; % rAmRBC

r(26) = Ksbache-ABache*KdachePls-KiachePLS*ABache*CPLS*FuPls + ABachei*KrachePLS; % rABache

r(27) = KiachePLS*ABache*CPLS*FuPls - ABachei*KrachePLS; % rABachei

r(28) = Ksbbche - ABbche*Kdbche - Kibche*ABbche*CPLS*FuPls + ABbchei*Krbche; % rABbche: rate bche

r(29) = Kibche*ABbche*CPLS*FuPls - ABbchei*Krbche; % rABbchei: bche inhibition, error corrected ABbchei*Krache->*Krbche

r(30) = kc*(AL+AVL+AF+AVF+AR+AVR+ABR+AVBR+APLS+AmRBC); % rTOTC

if mod(TIME,24) >= 12 && mod(TIME,24) < 12+TINF

r(31) = IVD/TINF; % rIV: IV infusion rate (umol/hr)

else

r(31) = 0;

end

r(15) = QC*(CV-CPLS)-r(27)-r(29)-r(17)-kc*APLS+r(31)-PARBC*(CPLS-CRBC/PRBC); % rAPLS: rate of carbaryl change in blood including iv dosing (umole/hr)

r(32) = 0;

dy = r;

end

end

***clip.m***

% Generate random numbers from a normal distribution

% Function to clip Monte Carlo distributions

% Generate random numbers from the normal distribution

% Cecilia Tan

% April 10, 2002

function normal = clip(mean,sd,ff)

ok = 0;

while ~ok

normal = normrnd(mean,sd); %Generates a random number from a normal distribution

ok = (normal>mean-ff*sd & normal<mean+ff*sd & normal>0); %Clip the distribution at mean +/- ff*standard deviation

end

***clipCV.m***

% Generate random numbers from a normal distribution

% Function to clip Monte Carlo distributions

% Generate random numbers from the normal distribution

% Cecilia Tan

% April 10, 2002

% CV should be in percentage (30% should be entered as 30, not 0.3)

function normal = clipCV(mean,CV,ff)

sd = mean*CV/100;

ok = 0;

while ~ok

normal = normrnd(mean,sd); %Generates a random number from a normal distribution

ok = (normal>mean-ff*sd & normal<mean+ff*sd & normal>0); %Clip the distribution at mean +/- ff*standard deviation

end

***initCarbaryl_param.m***

function P0 = initCarbaryl_param(Weight)

P0 = nan(64,1);

P0(1) = Weight; % BW; body weight kg

P0(2) = 0.45; % HCT; Hematocrit fraction

% VOLUMES (fraction of BW) Brown et al., 1997

P0(3) = 0.02; % VBRNC; brain

P0(4) = 0.21; % VFATC; fat

P0(5) = 0.026; % VLIVC; liver

P0(6) = 0.079; % VBLDC; blood

P0(7) = 0.05; % VTBC; fraction of tissue plasma (as multiples of tissue volume)

P0(8) = 0.85 - P0(3)-P0(4)-P0(5)-P0(6); % VBODC; excluding hard bone mass & non-perfused mass such GI content, seminal vesicle content, bladder content

% FLOWS (fraction of QC) Brown et al., 1997

P0(9) = 13; % QCC; cardiac output L/h/kg^0.75

P0(10) = 0.114; % QBRNC; brain

P0(11) = 0.052; % QFATC; fat

P0(12) = 0.227; % QLIVC; liver

P0(13) = 1 - P0(10) - P0(11) - P0(12); % QBODC

% *************** CARBARYL PBPK PARAMETERS ***************

% Distribution coefficients, tissue:blood (Experimentally determined eq dialysis)

P0(14) = 0.5; % PBRN; brain

P0(15) = 5.44; % PFAT; fat

P0(16) = 0.66; % PBOD; rest of body, muscle value

P0(17) = 1.15; % PLIV; liver

P0(18) = 0.78; % PRBC; RBC:plasma partition

% Fraction unbound in Tissues & blood

P0(19) = 0.17; % FuLiv;

P0(20) = 0.39; % FuBrn;

P0(21) = 0.2; % FuPls;

P0(22) = 0.25; % FuRBC;

% Tissue Permeability-area constants (L/h/kg^0.75) Nong et al posteriors

P0(23) = 0.83; % PAFC; fat

P0(24) = 6.29; % PARC; rest of body

P0(25) = 1.82; % PALC; liver

P0(26) = 0.13; % PABC; brain, diffusion limited

P0(27) = 100.; % PARBC;

% Biochemical parameters, from in vitro data

P0(28) = 22; % VKM1C; CL for carbaryl to other metabolites (L/hr/kg liver)

P0(29) = 0; % Vmax2C; Vmax for carbaryl to 1-naphthol (umol/hr/kg liver)

P0(30) = 34; % Km2; M-M constant for carbaryl to 1-naphthol (uM) !!! 34

P0(31) = 0; % VMax3C; Vmax for 1-naphthol sulfation (umol/hr/kg liver)

P0(32) = 1.; % Km3; uM

P0(33) = 1.; % Ki3; uM

P0(34) = 0; % Vmax4C; Vmax for 1-naphthol to other 1-napthol metabolites (umol/hr/kg liver)

P0(35) = 1; % Km4; uM

P0(36) = 0.0428; % kc; /hr

P0(37) = 23.7; % VKM2C; CL for carbaryl to 1-naphthol in plasma (L/hr/kg plasma)

% GI absorption constants

P0(38) = 4.0; % Ka; /hr, 1st order absorption rate, GI to liver (Houston et al., 1974)

P0(39) = 0; % KBileC; first order biliary excretion scalar (/hr/kg^-0.25)

P0(40) = 0.25; % Fa; fraction absorbed to systemic circulation

P0(41) = 0.2; % Fm; fraction of unabsorbed carbaryl absorbed as 1-naphthol

% *************** 1- Compartment for 1-naphthol ***************

% Biochemical

P0(42) = 0.2; % KenBC; urinary elimination of 1- naphthol and its metabolites (/hr/kg^-0.25)

%********* Added parameters for Urinary and Exposure **********

P0(43) = 1.; % VPEE; Volume of urine void

P0(44) = 1.; % LAST_VOID; Time of last void in hours

P0(45) = 1.; % FLOW_RATE; Flow rate

% ChE parameters

P0(46) = 462962; % BRACHE; Biegon & Wolff similar AChE level (ng/mg protein) by histochmistry postmortem brain rat vs human

P0(47) = 1; % BACHE; no AChE in human plasma

P0(48) = 99760; % BBCHE;

P0(49) = 395740; % RBACHE;

P0(50) = 1.17e+07; % TRCE;

P0(51) = 3.66e+06; % TRBE;

P0(52) = 3.708; % KIACHERBC;

P0(53) = 2.014; % KRACHERBC;

P0(54) = 3.708; % KIACHEBRN;

P0(55) = 2.014; % KRACHEBRN;

P0(56) = 1; % KIACHEPLS; no AChE in human plasma

P0(57) = 1; % KRACHEPLS; no AChE in human plasma

P0(58) = 0.01; % KDACHERBC;

P0(59) = 0.01; % KDACHEBRN;

P0(60) = 0.1; % KDACHEPLS;

P0(61) = 0.198; % KIBCHE;

P0(62) = 0.1; % KDBCHE;

P0(63) = 1.189; % KRBCHE;

P0(64) = 0; % IVDOSE; IV dose (ug/kg bw)

***initCarbaryl_param_MC.m***

function P0 = initCarbaryl_param_MC(Weight)

ff = 1.96;

P0 = nan(64,1);

P0(1) = Weight; % BW; body weight kg

P0(2) = clip(0.45,0.45*0.3,ff); % HCT; Hematocrit fraction

% VOLUMES (fraction of BW) Brown et al., 1997

P0(3) = clipCV(0.02,30,ff); % VBRNC; brain

P0(4) = clipCV(0.21,30,ff); % VFATC; fat

P0(5) = clipCV(0.026,30,ff); % VLIVC; liver

P0(6) = clipCV(0.079,30,ff); % VBLDC; blood

P0(7) = 0.05; % VTBC; fraction of tissue plasma (as multiples of tissue volume)

P0(8) = 0.85 - P0(3)-P0(4)-P0(5)-P0(6); % VBODC; excluding hard bone mass & non-perfused mass such GI content, seminal vesicle content, bladder content

% FLOWS (fraction of QC) Brown et al., 1997

P0(9) = clipCV(13,30,ff); % QCC; cardiac output L/h/kg^0.75

P0(10) = clipCV(0.114,30,ff); % QBRNC; brain

P0(11) = clipCV(0.052,30,ff); % QFATC; fat

P0(12) = clipCV(0.227,30,ff); % QLIVC; liver

P0(13) = 1 - P0(10) - P0(11) - P0(12); % QBODC

% *************** CARBARYL PBPK PARAMETERS ***************

% Distribution coefficients, tissue:blood (Experimentally determined eq dialysis)

P0(14) = clip(0.5,0.06,ff); % PBRN; brain

P0(15) = logclip(5.44,1.5,ff); % PFAT; fat

P0(16) = clip(0.66,0.14,ff); % PBOD; rest of body, muscle value

P0(17) = clip(1.15,0.05,ff); % PLIV; liver

P0(18) = clip(0.78,0.06,ff); % PRBC; RBC:plasma partition

% Fraction unbound in Tissues & blood

P0(19) = clip(0.17,0.01,ff); % FuLiv;

P0(20) = clip(0.39,0.04,ff); % FuBrn;

P0(21) = clip(0.2,0.01,ff); % FuPls;

P0(22) = clip(0.25,0.02,ff); % FuRBC;

% Tissue Permeability-area constants (L/h/kg^0.75) Nong et al posteriors

P0(23) = logclip(0.83,1.9,ff); % PAFC; fat

P0(24) = 6.29; % PARC; rest of body

P0(25) = 1.82; % PALC; liver

P0(26) = 0.13; % PABC; brain, diffusion limited

P0(27) = 100.; % PARBC;

% Biochemical parameters, from in vitro data

P0(28) = clipCV(22,88,ff); % VKM1C; CL for carbaryl to other metabolites (L/hr/kg liver)

P0(29) = 0; % Vmax2C; Vmax for carbaryl to 1-naphthol (umol/hr/kg liver)

P0(30) = 34; % Km2; M-M constant for carbaryl to 1-naphthol (uM) !!! 34

P0(31) = 0; % VMax3C; Vmax for 1-naphthol sulfation (umol/hr/kg liver)

P0(32) = 1.; % Km3; uM

P0(33) = 1.; % Ki3; uM

P0(34) = 0; % Vmax4C; Vmax for 1-naphthol to other 1-napthol metabolites (umol/hr/kg liver)

P0(35) = 1; % Km4; uM

P0(36) = clipCV(0.0428,30,ff); % kc; /hr

P0(37) = clipCV(23.7,24,ff); % VKM2C; CL for carbaryl to 1-naphthol in plasma (L/hr/kg plasma)

% GI absorption constants

P0(38) = 4.0; % Ka; /hr, 1st order absorption rate, GI to liver (Houston et al., 1974)

P0(39) = 0; % KBileC; first order biliary excretion scalar (/hr/kg^-0.25)

P0(40) = clipCV(0.25,50,ff); % Fa; fraction absorbed to systemic circulation

P0(41) = clipCV(0.2,50,ff); % Fm; fraction of unabsorbed carbaryl absorbed as 1-naphthol

% *************** 1- Compartment for 1-naphthol ***************

% Biochemical

P0(42) = 0.2; % KenBC; urinary elimination of 1- naphthol and its metabolites (/hr/kg^-0.25)

%********* Added parameters for Urinary and Exposure **********

P0(43) = 1.; % VPEE; Volume of urine void

P0(44) = 1.; % LAST_VOID; Time of last void in hours

P0(45) = 1.; % FLOW_RATE; Flow rate

% ChE parameters

P0(46) = 462962; % BRACHE; Biegon & Wolff similar AChE level (ng/mg protein) by histochmistry postmortem brain rat vs human

P0(47) = 1; % BACHE; no AChE in human plasma

P0(48) = 99760; % BBCHE;

P0(49) = 395740; % RBACHE;

P0(50) = 1.17e+07; % TRCE;

P0(51) = 3.66e+06; % TRBE;

P0(52) = clip(3.708,1.56,ff); % KIACHERBC;

P0(53) = clip(2.014,0.55,ff); % KRACHERBC;

P0(54) = clip(3.708,1.56,ff); % KIACHEBRN;

P0(55) = clip(2.014,0.55,ff); % KRACHEBRN;

P0(56) = 1; % KIACHEPLS; no AChE in human plasma

P0(57) = 1; % KRACHEPLS; no AChE in human plasma

P0(58) = clipCV(0.01,50,ff); % KDACHERBC;

P0(59) = clipCV(0.01,50,ff); % KDACHEBRN;

P0(60) = 0.1; % KDACHEPLS;

P0(61) = 0.198; % KIBCHE;

P0(62) = 0.1; % KDBCHE;

P0(63) = 1.189; % KRBCHE;

P0(64) = 0; % IVDOSE; IV dose (ug/kg bw)

***logclip.m***

% Generate random numbers for a lognormal distribution

% Function to clip Monte Carlo distributions

% The conversion of O and P to M and V, where

% U is normally distributed with mean (M) and variance (V)

% If U = ln (Y), then Y is lognormally distributed with mean (O) and variance (P)

% O = exp(M+0.5*V)

% P = exp(2M+2V)-exp(2M+V)

%

% M = ln(O) – 0.5*ln(P/O^2 +1)

% V = ln(P/O^2 +1)

% stdev=sqrt(V)

%

% Input: mean (O), SD (sqrt(P)), and # SD for cut-off (ff)

function lognorm = logclip(O,SD,ff)

M = log(O)-0.5*log(1+SD^2./O^2);

V = log(1+SD^2./O^2);

stdev = sqrt(V);

UB = exp(M + ff*stdev);

LB = exp(M - ff*stdev);

within = 0;

while ~within

lognorm = lognrnd(M,stdev); %Generates a random number from a lognormal distribution

within = (lognorm>LB & lognorm<UB & lognorm>0); %Clip the distribution within mean +/- ff*standard dev.

end

***runScript.m***

function simulationOutput = runScript(personNumber,filtered,avgDailyDose,dailyDoses,Weight,FlowRate)

totPerson = 500;

multiplier = 10;

out1 = nan(totPerson,multiplier);

out2 = nan(totPerson,multiplier);

out3 = nan(totPerson,multiplier);

out4 = nan(totPerson,multiplier);

out5 = nan(totPerson,multiplier);

out6 = nan(totPerson,multiplier);

out7 = nan(totPerson,multiplier);

out8 = nan(totPerson,multiplier);

out9 = nan(totPerson,multiplier);

out10 = nan(totPerson,multiplier);

out11 = nan(totPerson,multiplier);

out12 = nan(totPerson,multiplier);

out13 = nan(totPerson,multiplier);

parfor nPerson = 1:totPerson

startIndex = find(personNumber == nPerson);

if nPerson ~= 500

stopIndex = find(personNumber == nPerson+1)-1;

else

stopIndex = length(personNumber);

end

totExposures = stopIndex-startIndex+1;

Exposure = nan(totExposures,2);

for nExposure = 1:totExposures

Exposure(nExposure,1) = filtered(startIndex+nExposure-1, 2)/60 + 24*(filtered(startIndex+nExposure-1, 1)-filtered(1,1));

Exposure(nExposure,2) = filtered(startIndex+nExposure-1, 3)*1000;

end

for mult = 1:multiplier

out = carbarylPBPK(Exposure,Weight(nPerson));

ok = 0;

nDays = max(filtered(:,1)) - min(filtered(:,1));

while ~ok

randSample1 = rand*24 + nDays*24;

randSample2 = rand*24 + nDays*24;

ok = abs(randSample1 - randSample2) > 1;

end

if randSample1 < randSample2

lastVoid = randSample1;

sampleTime = randSample2;

else

lastVoid = randSample2;

sampleTime = randSample1;

end

lastDay = sampleTime-24;

matchVoidTime = (out.Time-lastVoid).^2;

[~,urineStart] = min(matchVoidTime);

matchSampleTime = (out.Time-sampleTime).^2;

[~,spotTime] = min(matchSampleTime);

voidVolume = (sampleTime - lastVoid)*FlowRate(nPerson,1);

matchLastDay = (out.Time-lastDay).^2;

[~,startOfLastDay] = min(matchLastDay);

matchBegin24 = (Exposure(:,1)-lastDay);

matchEnd24 = (Exposure(:,1)-sampleTime);

indexExposureBegin = find(matchBegin24 > 0,1,'first');

indexExposureEnd = find(matchEnd24 < 0,1,'last');

out1(nPerson,mult) = avgDailyDose(nPerson,1); % Average daily dose for a year

out2(nPerson,mult) = (out.UClNPall(spotTime,1)-out.UClNPall(urineStart,1))/voidVolume; % 1-N urine concentration

out3(nPerson,mult) = out.PRBCdep(spotTime,1); % RBC AChE depression (%)

out4(nPerson,mult) = out.PBRNdep(spotTime,1); % Brain AChE depression (%)

out5(nPerson,mult) = mean(out.ABR(startOfLastDay:spotTime,1))/((0.02*70)*(1-0.05)); % 24 hr avg brain concentration

out6(nPerson,mult) = mean(dailyDoses(nPerson,359:365)); % Average daily dose for the last week

out7(nPerson,mult) = dailyDoses(nPerson,365); % Average daily dose for the last day of dosing

out8(nPerson,mult) = out.CPlasma(spotTime,1); % Spot plasma concentration

out9(nPerson,mult) = mean(dailyDoses(nPerson,364:365)); % Average daily dose for the last two days of dosing

out10(nPerson,mult) = mean(out.PRBCdep(startOfLastDay:spotTime,1)); % Average RBC AChE depression over the final 24 hrs

out11(nPerson,mult) = mean(out.PBRNdep(startOfLastDay:spotTime,1)); % Average Brain AChE depression over the final 24 hrs

out12(nPerson,mult) = Exposure(indexExposureEnd,2); % magnitude of the last exposure before sampling

out13(nPerson,mult) = sum(Exposure(indexExposureBegin:indexExposureEnd,2)); % Exposure in the final 24 hours

end

end

out1 = reshape(out1,totPerson*multiplier,1);

out2 = reshape(out2,totPerson*multiplier,1);

out3 = reshape(out3,totPerson*multiplier,1);

out4 = reshape(out4,totPerson*multiplier,1);

out5 = reshape(out5,totPerson*multiplier,1);

out6 = reshape(out6,totPerson*multiplier,1);

out7 = reshape(out7,totPerson*multiplier,1);

out8 = reshape(out8,totPerson*multiplier,1);

out9 = reshape(out9,totPerson*multiplier,1);

out10 = reshape(out10,totPerson*multiplier,1);

out11 = reshape(out11,totPerson*multiplier,1);

out12 = reshape(out12,totPerson*multiplier,1);

out13 = reshape(out13,totPerson*multiplier,1);

simulationOutput = nan(totPerson*multiplier,13);

simulationOutput(:,1) = out1(:,1);

simulationOutput(:,2) = out2(:,1);

simulationOutput(:,3) = out3(:,1);

simulationOutput(:,4) = out4(:,1);

simulationOutput(:,5) = out5(:,1);

simulationOutput(:,6) = out6(:,1);

simulationOutput(:,7) = out7(:,1);

simulationOutput(:,8) = out8(:,1);

simulationOutput(:,9) = out9(:,1);

simulationOutput(:,10) = out10(:,1);

simulationOutput(:,11) = out11(:,1);

simulationOutput(:,12) = out12(:,1);

simulationOutput(:,13) = out13(:,1);

***sensitivityAnalysis.m***

function [sensitivityCoefficients,SC_timecourses] = sensitivityAnalysis(parameterNames)

truncTime = 168;

coeff_UClNPall = nan(64,3);

coeff_PRBCdep = nan(64,3);

coeff_PBRNdep = nan(64,3);

coeff_ABR = nan(64,3);

coeff_CPlasma = nan(64,3);

list_UClNPall = zeros(64,3);

list_PRBCdep = zeros(64,3);

list_PBRNdep = zeros(64,3);

list_ABR = zeros(64,3);

list_CPlasma = zeros(64,3);

final_UClNPall(64,3) = {NaN};

final_PRBCdep(64,3) = {NaN};

final_PBRNdep(64,3) = {NaN};

final_ABR(64,3) = {NaN};

final_CPlasma(64,3) = {NaN};

Weight = 72;

P0 = initCarbaryl_param(Weight);

for i = 1:3

switch i

case 1

dose = 2.9670e-05*1000;

case 2

dose = 4.2606e-04*1000;

case 3

dose = 4.2606e-04*1000*1000;

end

Exposures = [12,dose;36,dose;60,dose;84,dose;108,dose;132,dose;156,dose;168,0;180,dose];

out_base = carbarylPBPK_forSA(Exposures,Weight,P0);

allTimesT_base = out_base.Time;

matchTime = (allTimesT_base-truncTime).^2;

[~,truncateHere] = min(matchTime);

t_base = allTimesT_base(truncateHere:end,1);

UClNPall_base = out_base.UClNPall(truncateHere:end,1);

PRBCdep_base = out_base.PRBCdep(truncateHere:end,1);

PBRNdep_base = out_base.PBRNdep(truncateHere:end,1);

ABR_base = out_base.ABR(truncateHere:end,1);

CPlasma_base = out_base.CPlasma(truncateHere:end,1);

t_base_ten = t_base*10;

t_base_ten = round(t_base_ten);

[t_base, keepThese, ~] = unique(t_base_ten);

t_base = t_base/10;

UClNPall_base = UClNPall_base(keepThese);

PRBCdep_base = PRBCdep_base(keepThese);

PBRNdep_base = PBRNdep_base(keepThese);

ABR_base = ABR_base(keepThese);

CPlasma_base = CPlasma_base(keepThese);

parfor j = 2:64

ok = j ~= 8 && j ~= 13

if ok == 1;

P0_low = P0;

P0_low(j) = P0_low(j)-0.005*P0_low(j);

P0_high = P0;

P0_high(j) = P0_high(j)+0.005*P0_high(j);

out_low = carbarylPBPK_forSA(Exposures,Weight,P0_low);

allTimesT_low = out_low.Time;

matchTime_low = (allTimesT_low-truncTime).^2;

[~,truncateHere_low] = min(matchTime_low);

t_low = allTimesT_low(truncateHere_low:end,1);

UClNPall_low = out_low.UClNPall(truncateHere_low:end,1);

PRBCdep_low = out_low.PRBCdep(truncateHere_low:end,1);

PBRNdep_low = out_low.PBRNdep(truncateHere_low:end,1);

ABR_low = out_low.ABR(truncateHere_low:end,1);

CPlasma_low = out_low.CPlasma(truncateHere_low:end,1);

t_low_ten = t_low*10;

t_low_ten = round(t_low_ten);

[t_low, keepThese_low, ~] = unique(t_low_ten);

t_low = t_low/10;

UClNPall_low = UClNPall_low(keepThese_low);

PRBCdep_low = PRBCdep_low(keepThese_low);

PBRNdep_low = PBRNdep_low(keepThese_low);

ABR_low = ABR_low(keepThese_low);

CPlasma_low = CPlasma_low(keepThese_low);

out_high = carbarylPBPK_forSA(Exposures,Weight,P0_high);

allTimesT_high = out_high.Time;

matchTime_high = (allTimesT_high-truncTime).^2;

[~,truncateHere_high] = min(matchTime_high);

t_high = allTimesT_high(truncateHere_high:end,1);

UClNPall_high = out_high.UClNPall(truncateHere_high:end,1);

PRBCdep_high = out_high.PRBCdep(truncateHere_high:end,1);

PBRNdep_high = out_high.PBRNdep(truncateHere_high:end,1);

ABR_high = out_high.ABR(truncateHere_high:end,1);

CPlasma_high = out_high.CPlasma(truncateHere_high:end,1);

t_high_ten = t_high*10;

t_high_ten = round(t_high_ten);

[t_high, keepThese_high, ~] = unique(t_high_ten);

t_high = t_high/10;

UClNPall_high = UClNPall_high(keepThese_high);

PRBCdep_high = PRBCdep_high(keepThese_high);

PBRNdep_high = PBRNdep_high(keepThese_high);

ABR_high = ABR_high(keepThese_high);

CPlasma_high = CPlasma_high(keepThese_high);

if t_low == t_high & t_low == t_base

normSC_UClNPall = (UClNPall_high-UClNPall_low)./(0.01*UClNPall_base);

normSC_PRBCdep = (PRBCdep_high-PRBCdep_low)./(0.01*PRBCdep_base);

normSC_PBRNdep = (PBRNdep_high-PBRNdep_low)./(0.01*PBRNdep_base);

normSC_ABR = (ABR_high-ABR_low)./(0.01*ABR_base);

normSC_CPlasma = (CPlasma_high-CPlasma_low)./(0.01*CPlasma_base);

coeff_UClNPall(j,i) = mean(normSC_UClNPall);

coeff_PRBCdep(j,i) = mean(normSC_PRBCdep);

coeff_PBRNdep(j,i) = mean(normSC_PBRNdep);

coeff_ABR(j,i) = mean(normSC_ABR);

coeff_CPlasma(j,i) = mean(normSC_CPlasma);

final_UClNPall{j,i} = normSC_UClNPall;

final_PRBCdep{j,i} = normSC_PRBCdep;

final_PBRNdep{j,i} = normSC_PBRNdep;

final_ABR{j,i} = normSC_ABR;

final_CPlasma{j,i} = normSC_CPlasma;

if median(abs(normSC_UClNPall)) > 0.1

list_UClNPall(j,i) = 1;

else

if max(normSC_UClNPall) > 0.1

list_UClNPall(j,i) = 2;

%out_a{j,i} = normSC_amount;

end

end

if median(abs(normSC_PRBCdep)) > 0.1

list_PRBCdep(j,i) = 1;

else

if max(normSC_PRBCdep) > 0.1

list_PRBCdep(j,i) = 2;

%out_r{j,i} = normSC_rate;

end

end

if median(abs(normSC_PBRNdep)) > 0.1

list_PBRNdep(j,i) = 1;

else

if max(normSC_PBRNdep) > 0.1

list_PBRNdep(j,i) = 2;

%out_r{j,i} = normSC_rate;

end

end

if median(abs(normSC_ABR)) > 0.1

list_ABR(j,i) = 1;

else

if max(normSC_ABR) > 0.1

list_ABR(j,i) = 2;

%out_r{j,i} = normSC_rate;

end

end

if median(abs(normSC_CPlasma)) > 0.1

list_CPlasma(j,i) = 1;

else

if max(normSC_CPlasma) > 0.1

list_CPlasma(j,i) = 2;

%out_r{j,i} = normSC_rate;

end

end

else

error('sensitivityAnalysis:MatchError','Add more code - times are not matching')

end

end

end

end

sensitivityCoefficients.UClNPall = coeff_UClNPall;

sensitivityCoefficients.PRBCdep = coeff_PRBCdep;

sensitivityCoefficients.PBRNdep = coeff_PBRNdep;

sensitivityCoefficients.ABR = coeff_ABR;

sensitivityCoefficients.CPlasma = coeff_CPlasma;

format compact

for m = 1:3

switch m

case 1

disp('Low Dose 0.0297 mg/kg/day')

case 2

disp('High Dose 0.4261 mg/kg/day')

case 3

disp('Ultra High Dose 426.1 mg/kg/day')

end

for n = 1:5

switch n

case 1

disp('Sensitivity of Urinary 1-N Concentration')

case 2

disp('Sensitivity of RBC AChE Depression')

case 3

disp('Sensitivity of Brain AChE Depression')

case 4

disp('Sensitivity of Brain Carbaryl Concentration')

case 5

disp('Sensitivity of Plasma Carbaryl Concentration')

end

for o = 1:2

switch o

case 1

disp('Sensitive Parameters')

for p = 1:64

if n == 1

if list_UClNPall(p,m) == 1

disp(parameterNames{p,1})

end

elseif n == 2

if list_PRBCdep(p,m) == 1

disp(parameterNames{p,1})

end

elseif n == 3

if list_PBRNdep(p,m) == 1

disp(parameterNames{p,1})

end

elseif n == 4

if list_ABR(p,m) == 1

disp(parameterNames{p,1})

end

elseif n == 5

if list_CPlasma(p,m) == 1

disp(parameterNames{p,1})

end

end

end

case 2

disp('Borderline Sensitive Parameters')

for p = 1:64

if n == 1

if list_UClNPall(p,m) == 2

disp(parameterNames{p,1})

end

elseif n == 2

if list_PRBCdep(p,m) == 2

disp(parameterNames{p,1})

end

elseif n == 3

if list_PBRNdep(p,m) == 2

disp(parameterNames{p,1})

end

elseif n == 4

if list_ABR(p,m) == 2

disp(parameterNames{p,1})

end

elseif n == 5

if list_CPlasma(p,m) == 2

disp(parameterNames{p,1})

end

end

end

end

end

end

end

consolidatedList = zeros(64,1);

for q = 1:64

if list_UClNPall(q,1) == 2 || list_UClNPall(q,2) == 2 || list_UClNPall(q,3) == 2 || list_PRBCdep(q,1) == 2 || list_PRBCdep(q,2) == 2 || list_PRBCdep(q,3) == 2 || list_PBRNdep(q,1) == 2 || list_PBRNdep(q,2) == 2 || list_PBRNdep(q,3) == 2 || list_ABR(q,1) == 2 || list_ABR(q,2) == 2 || list_ABR(q,3) == 2 || list_CPlasma(q,1) == 2 || list_CPlasma(q,2) == 2 || list_CPlasma(q,3) == 2

consolidatedList(q,1) = 1;

end

end

indices = find(consolidatedList);

nEntries = sum(consolidatedList);

SC_timecourses(nEntries,15) = {NaN};

for thisEntry = 1:nEntries

index = indices(thisEntry);

SC_timecourses{thisEntry,1} = final_UClNPall{index,1};

SC_timecourses{thisEntry,2} = final_UClNPall{index,2};

SC_timecourses{thisEntry,3} = final_UClNPall{index,3};

SC_timecourses{thisEntry,4} = final_PRBCdep{index,1};

SC_timecourses{thisEntry,5} = final_PRBCdep{index,2};

SC_timecourses{thisEntry,6} = final_PRBCdep{index,3};

SC_timecourses{thisEntry,7} = final_PBRNdep{index,1};

SC_timecourses{thisEntry,8} = final_PBRNdep{index,2};

SC_timecourses{thisEntry,9} = final_PBRNdep{index,3};

SC_timecourses{thisEntry,10} = final_ABR{index,1};

SC_timecourses{thisEntry,11} = final_ABR{index,2};

SC_timecourses{thisEntry,12} = final_ABR{index,3};

SC_timecourses{thisEntry,13} = final_CPlasma{index,1};

SC_timecourses{thisEntry,14} = final_CPlasma{index,2};

SC_timecourses{thisEntry,15} = final_CPlasma{index,3};

end
